# Acute activation of adipocyte lipolysis reveals dynamic lipid remodeling of the hepatic lipidome

**DOI:** 10.1016/j.jlr.2023.100434

**Published:** 2023-08-26

**Authors:** Sicheng Zhang, Kevin J. Williams, Amandine Verlande-Ferrero, Alvin P. Chan, Gino B. Su, Erin E. Kershaw, James E. Cox, John Alan Maschek, Suzanne N. Shapira, Heather R. Christofk, Thomas Q. de Aguiar Vallim, Selma Masri, Claudio J. Villanueva

**Affiliations:** 1 Department of Integrative Biology and Physiology, University of California, Los Angeles (UCLA), Los Angeles, CA, USA; 2 UCLA Lipidomics Lab, Department of Biological Chemistry, David Geffen School of Medicine, University of California, Los Angeles (UCLA), Los Angeles, CA, USA; 3 Department of Biological Chemistry, Center for Epigenetics and Metabolism, Chao Family Comprehensive Cancer Center, University of California, Irvine (UCI), Irvine, CA, USA; 4 Department of Biological Chemistry, David Geffen School of Medicine, University of California, Los Angeles (UCLA), Los Angeles, CA, USA; 5 Division of Endocrinology and Metabolism, Department of Medicine, University of Pittsburgh, PA, USA; 6 Department of Biochemistry, University of Utah School of Medicine, Salt Lake City, UT, USA; 7 Nutrition and Integrative Physiology, College of Health, University of Utah, Salt Lake City, UT, USA; 8 Molecular Biology Institute, University of California, Los Angeles (UCLA), Los Angeles, CA, USA; 9 Division of Cardiology, Department of Medicine, University of California, Los Angeles (UCLA), Los Angeles, CA, USA

**Keywords:** Adipocytes, Adipose tissue triglyceride lipase, Lipase, Lipids, Liver, Ceramides, Triglycerides, Lipidomics, Lipid droplets, Fasting

## Abstract

Adipose tissue is the site of long-term energy storage. During the fasting state, exercise, and cold exposure, the white adipose tissue mobilizes energy for peripheral tissues through lipolysis. The mobilization of lipids from white adipose tissue to the liver can lead to excess triglyceride accumulation and fatty liver disease. Although the white adipose tissue is known to release free fatty acids, a comprehensive analysis of lipids mobilized from white adipocytes in vivo has not been completed. In these studies, we provide a comprehensive quantitative analysis of the adipocyte-secreted lipidome and show that there is interorgan crosstalk with liver. Our analysis identifies multiple lipid classes released by adipocytes in response to activation of lipolysis. Time-dependent analysis of the serum lipidome showed that free fatty acids increase within 30 min of β3-adrenergic receptor activation and subsequently decrease, followed by a rise in serum triglycerides, liver triglycerides, and several ceramide species. The triglyceride composition of liver is enriched for linoleic acid despite higher concentrations of palmitate in the blood. To further validate that these findings were a specific consequence of lipolysis, we generated mice with conditional deletion of adipose tissue triglyceride lipase exclusively in adipocytes. This loss of in vivo adipocyte lipolysis prevented the rise in serum free fatty acids and hepatic triglycerides. Furthermore, conditioned media from adipocytes promotes lipid remodeling in hepatocytes with concomitant changes in genes/pathways mediating lipid utilization. Together, these data highlight critical role of adipocyte lipolysis in interorgan crosstalk between adipocytes and liver.

The adipose tissue has tremendous metabolic plasticity and can expand during times of energy excess. Over time excess accumulation of lipids in the adipose tissue promotes obesity and can increase the risk for developing conditions like the metabolic syndrome (1). When energy demands are high in peripheral tissues, FFAs are released from adipocytes through the regulated process of lipolysis. Enhanced release of FFAs and other lipids from adipocytes contribute to triglyceride (TAG) accumulation in the liver, heart, and skeletal muscle, leading to conditions such as nonalcoholic fatty liver disease (NAFLD), diabetes, and cardiovascular disease (2, 3, 4). Remarkably, obesity is associated with an increased risk of NAFLD, and a rise in basal lipolysis has been implicated in mediating these effects.

Adipocyte lipolysis plays a critical role in the metabolic transition between the fed and fasted state, exercise, thermogenesis, and cancer cachexia (5, 6, 7, 8, 9, 10, 11, 12). Furthermore, adipocyte lipolysis is regulated by numerous pharmacological agents including anti-inflammatories, psychiatric medications, insulin, rosiglitazone, and glucocorticoids (13, 14, 15, 16). Several studies have examined the composition of adipose tissue or the adipocyte secretome (17, 18, 19). The adipocyte secretome includes lipids, proteins, hormones, RNAs, and other bioactive factors (9, 10, 12, 20, 21). Metabolites can act as metabolic precursors or as signaling molecules that provide important paracrine and endocrine communication during metabolic transitions. FFAs can provide substrate or act as signals to regulate lipid remodeling in multiple organs (9, 10, 22, 23). Although FAs have been implicated as signaling molecules, a comprehensive quantitative assessment and compositional analysis of products of lipolysis has not been determined. Furthermore, both the time-dependent changes in plasma lipids and lipid remodeling of the liver have not been assessed. During adipocyte lipolysis, a lipidome from a short time or single stimulation to adipocyte lipolysis might differ from other long-term physiological treatments or long-term adipocyte lipolysis stimulation (10). Therefore, revealing the signals or substrate from lipolysis would contribute to defining how white adipose tissue (WAT)-derived lipids change over time and the impact on hepatic lipids.

Physiological adaptations to conditions such as cold exposure or exercise stimulate adipocyte lipolysis through release of norepinephrine by the sympathetic nervous system (24, 25). Norepinephrine promotes the activation of β1, β2, and β3 adrenergic receptors (ARs) that are found on the plasma membrane of adipocytes. While the β1AR and β2AR are expressed in multiple tissues, the β3-AR has limited expression, which is confined to white and brown adipocytes. Small molecules such as CL-316,243 (CL) can be used to mimic lipolysis by activating β3ARs. Long-term treatment with CL promotes remodeling of both brown adipose tissue (BAT) and WAT browning (26, 27, 28). There are several enzymes involved in the complete hydrolysis of TAGs (29, 30, 31). The first step is completed by adipose tissue triglyceride lipase (ATGL), which hydrolyzes TAGs on the lipid droplet surface to generate an acyl chain and diacylglycerol (DAG) (32). Conditional deletion of ATGL in adipocytes prevents a rise in blood FFAs in response to CL-316,243 (11, 33).

There is evidence to suggest that lipid mediators promote chronic liver disease, such as NAFLD, nonalcoholic steatohepatitis, and hepatocellular carcinoma (34, 35, 36, 37). Use of stable isotope tracers suggests that NAFLD and obese patients have a greater amount of FFA release from adipose tissue into plasma, contributing to lipid accumulation in the liver (38, 39, 40). Adipocyte lipolysis releases a myriad of lipid species that could impact hepatic lipid remodeling and function. Given the profound impact of these processes on human health and disease, there is a tremendous need for a more granular and the context-specific understanding of adipocyte lipolysis on the serum and hepatic lipidome.

In this study, we applied quantitative lipidomic analysis to assess how lipids in circulation and the liver change over time after an acute stimulus of adipocyte lipolysis. This method provided a comprehensive coverage of over 1400 lipids species across 17 subclasses using a broadly targeted approach that provides a quantitative assessment of lipid species. With a combination of 70 lipid standards, we quantified the molar concentration of each specific lipid. Our analysis showed how dynamic changes in FAs impact other lipid classes like ceramides (CERs) and a variety of phospholipid species. Use of mice with conditional deletion of ATGL in adipocytes allowed us to directly interrogate how signals from adipose tissue can impact lipid remodeling in the liver. Highlighting the lipid species in serum that are a product of adipocyte lipolysis and how they drive lipid remodeling in the liver.

## Materials and Methods

### Animals and treatment

C57BL6J mice were acquired from the Jackson Laboratory (Bar, Harbor, ME, 000664). *Pnpla2*^*F/F*^ (Jackson Laboratory, 024278) were crossed with *Adipoq*^*CRE*^ mice (Jackson Laboratory, 028020). All animals were housed in a room with controlled temperature (20–24°C), a 12 h light dark cycle, and free access to food and water. All mice used for experiment were male mice. For CL-316,243 treatment, the mice were treated with a single dose of 1 mg/kg CL-316,243 by intraperitoneal injection. After injection, the mice were provided water but no food. Mice had free access to food prior to CL-316,243 administration. These studies were approved by the UCLA institutional animal care and use committee.

### Tissue culture

Pre-adipocytes were seeded at 1,000,000 cells in a 10 cm dish (preadipocytes immortalized with large T antigen) with Dulbecco's Modified Eagle Medium (DMEM) + 10% fetal bovine serum (FBS) + 5 μg/ml insulin). At confluence, cells were differentiated with DMEM + 10% FBS + DMI + GW1929 cocktail (1 μM dexamethasone (D), 0.5 mM IBMX (M), 5 μg/ml insulin (I), 20 nM GW1929). After 2 days, media replaced with DMEM + 10% FBS + 5 μg/ml insulin + 20 nM GW1929 and replaced with fresh DMEM + 10% FBS + 5 μg/ml insulin + 20 nM GW1929 every 2 days until day 10.

Lipolysis assay: adipocytes differentiated for 10 days were incubated with Krebs Ringer buffer for 1 h, media then replaced with or without 100 nM CL-316,243 or phosphate buffered saline, and incubated for 5 h before media was collected.

Hepa1-6 was cultured in DMEM + 10% FBS. For condition medium (CM) treatment, confluent Hepa1-6 hepatocytes were treated with ringer buffer for 1 h. Then, the Hepa1-6 cells were treated with CM for 5 h.

Hepatocyte organoids differentiation and culture were described previously (41). Organoids were treated with CM for 5 h.

### Tissue RNA isolation and quantitative real-time PCR

Total RNA of mouse liver tissue was isolated using TRIzol reagent (Invitrogen, Carlsbad, CA). Complimentary deoxyribonucleid acid library was prepared by High-Capacity complimentary deoxyribonucleid acid reverse transcription kit (Catalog: 4368814, Thermo Fisher Scientific). Gene expression quantification was performed with KAPA SYBR FAST qPCR 2x Master Mix Lightcycle (Catalog: 7959591001, Kapa Biosystems) on an Applied Biosystems QuantStudio 6 Flex Real-Time PCR System. Quantification completed using standard curve method.

### Lipid extraction and lipidomic analysis

25 μl serum, 2–6 mg homogenized tissue, or 0.5 ml culture media used for lipidomic analysis. For homogenized tissue, 50–100 mg of tissue were collected in a 2 ml homogenizer tube preloaded with 2.8 mm ceramic beads (Omni #19-628). 0.75 ml PBS was added to the tube and homogenized in the Omni Bead Ruptor Elite (3 cycles of 10 s at five m/s with a 10 s dwell time).

Homogenate containing 2–6 mg of original tissue was transferred to a glass tube for extraction. A modified Bligh and Dyer extraction (42) was carried out on all samples. Prior to biphasic extraction, a standard internal mixture consisting of 70 lipid standards across 17 subclasses was added to each sample (AB Sciex 5040156, Avanti 330827, Avanti 330830, Avanti 330828, Avanti 791642). (Note only AB Sciex 5040156 used in early experiments).

Following two successive extractions, pooled organic layers were dried down in a Thermo SpeedVac SPD300DDA using a ramp setting 4 at 35 degrees C for 45 min with a total run time of 90 min.

Lipid samples were resuspended in 1:1 methanol/dichloromethane with 10 mM ammonium acetate and transferred to robovials (Thermo Fisher Scientific, 10800107) for analysis.

Samples were analyzed on the Sciex 5500 with DMS device (Lipidyzer Platform) with an expanded targeted acquisition list consisting of 1450 lipid species across 17 subclasses (or the original acquisition list of 1100 lipids across 13 subclasses). Differential mobility device on Lipidyzer was tuned with EquiSPLASH LIPIDOMIX (Avanti 330731). Data analysis performed on an in-house data analysis platform comparable to the Lipidyzer Workflow Manager. Instrument method including settings, tuning protocol, and MRM list is available in Su *et al.* Quantitative values were normalized to plasma/media volume or mg of tissue.

### Metabolites extraction and LC-MS

25 μl serum or 25 μL culture media used for metabolites analysis. Add 500 µL cold MeOH to each tube, vortex, and wait 1 min. Add 200 µL Milli-Q water containing norvaline to each tube. Add 500 µL chloroform to each vial. Centrifuge samples for 6 min at 10,000 *g* at 4°C. Keep the upper MeOH/H2O layer containing polar metabolites. Evaporate each sample.

### Indirect calorimetry and isotope analysis of ^13^CO_2_

Oxygen consumption (ml/h), energy expenditure (EE, kcal/h), and respiratory exchange ratio (RER) were monitored for individually housed WT mice using the Phenomaster metabolic cages (TSE Systems Inc., Chesterfield, MO). The climate chamber was set to 21°C, 50% humidity, with a 12:12 light-dark cycle as the home-cage environment. Data were collected at 22-min intervals, and each cage was recorded for 2.75 min before time point collection. Mice were food-restricted during the stable isotope experiment, which was performed in the light phase. Mice were gavaged once with U-C13 palmitic acid (Catalog: CLM-409-0.5) or U-C13 linoleic acid (CLM-6855-0.25), followed by intraperitoneal injection an hour later with saline or 1 mg/kg CL-316,243. Exhaled ^13^CO2 for each cage was normalized to total CO2 abundance to quantify changes in systemic palmitic acid or linoleic acid utilization.

### Quantification of ^13^C-labeled FAs by GC-MS

FA methyl esters were extracted from liver and serum samples using mild acid methanolysis, which included a mixture of hydrochloric acid/methanol/toluene supplemented with triheptadecenoin (Nu-Chek Prep, T-404) as an internal standard. FA methyl esters were measured by GC-MS using an Agilent 7890B/5977A. Both labeled (^13^C) and unlabeled (^12^C) linoleic acid and palmitic acid methyl esters were quantified based on standards consisting of serial dilutions of ^13^C-palmitate (Cambridge Isotope Laboratories, CLM-409), ^13^C-linoleate (Cambridge Isotope Laboratories, CLM-6855), and a standard FA mix containing ^12^C-palmitate and ^12^C-linoleate (Nu-Chek Prep, GLC-96). Incorporation of ^13^C-labeled palmitate and linoleate in liver and serum was calculated as a ratio of the respective ^13^C-labeled to total ^12^C and ^13^C-labeled FA.

### Analysis of lipidome from serum and liver tissue

The lipidome data pie and bar graphs were plotted by Prism 7. The heat map was performed by R language pheatmap (clustered by R cluster WardD.2 or Ward).

## Results

### Targeted quantitative lipidomic analysis using LC-MS of whole mouse serum after acute activation of adipocyte lipolysis

**Fig. 1:**
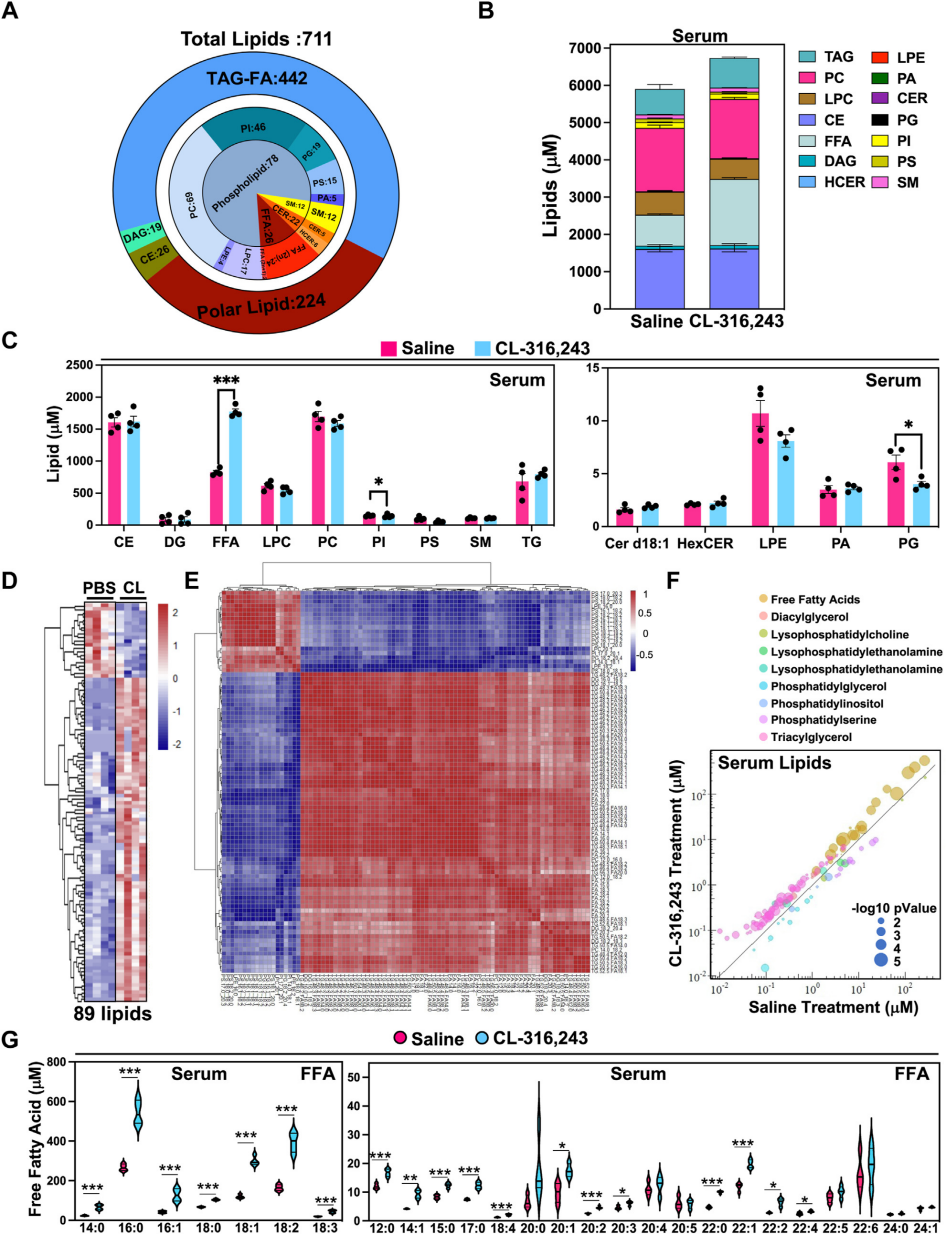
Quantitative lipidomic analysis of serum with acute activation of adipose tissue lipolysis. A: Graphical representation of lipids identified in mouse serum after 30 min of CL-316,243 administration grouped by the corresponding lipid class. B: Concentration of lipid molecular species after 30 min of saline or CL administration grouped by the corresponding lipid class. C: Major lipid classes in serum 30 min with saline or CL-316,243 administration. D: Heatmap highlighting significant lipid changes between saline of CL-316,243 administration (n = 4, *P* < 0.05). E: Correlation cluster of 89 significant lipids. F: Distribution of significant lipids by lipid classes, concentration, and *P*-value. Color represents lipids classes, Y axis represents the lipid concentration after CL treatment, X axis represents saline treatment. Dot diameter represents –log10 (*P*-value). G: Violin plots highlighting compositional changes of serum FFA species with saline or 30 min of CL administration. (n = 4, ∗: *P* < 0.05, ∗∗: *P* < 0.01, ∗∗∗: *P* < 0.005).

To systematically quantify lipids in serum upon activation of lipolysis, we applied a comprehensive quantitative approach to analyze lipids. Previously we completed a time course to understand how FFAs change during cold exposure in mice and found that serum FFAs peak at 30 min. Therefore, we used the 30-min time point with a single dose of 1 mg/kg of CL-316,243 (CL), a beta3-AR agonist to provide a thorough quantitative analysis of both serum and hepatic lipid changes (Fig. 1 and supplemental Fig. S2D, E). Using a LC-MS/MS approach, we were able to assess the concentrations of 1450 lipids with corresponding standards that can provide a quantitative assessment of the serum lipidome (43). Through this analysis, we identified 771 lipid species that fell under 17 lipid classes (Fig. 1A). Lipids were grouped by their corresponding lipid class, which was primarily composed of TAGs and polar lipids, with fewer lipids under the category of DAG and cholesteryl esters (CE). Polar lipids include FAs (FFAs), SMs, CERs, and several phospholipid species (Fig. 1B). All detailed specific lipids change has been shown in supplemental Fig. S1. We found that FFAs were the only lipids to increase in response to CL administration (Fig. 1B, C). In contrast, we found that PI and phosphatidylglycerol decreased during this time (Fig. 1C). Of the 771 lipids quantified in serum, we found 89 lipids that significantly change in response to CL (Fig. 1D–F). Correlation cluster analysis of serum lipids suggest that serum FFAs and serum TG are coregulated (Fig. 1E). Most of these lipids were FAs with varying lengths and saturation. Notably, there were a few TAG species that were elevated in response to CL (Fig. 1F).

Further analysis showed that various FFA species were elevated in response to CL, where the most abundant were myristic acid (C14:0), palmitic acid (C16:0), palmitoleic acid (C16:0), stearic acid (C18:0), oleic acid (C18:1), linoleic acid (C18:2), and linolenic acid (C18:3). However, several very-long-chain FAs (VLCFAs) did not change, such as docosahexaenoic acid (C22:6), lignoceric acid (C24:0), nervonic acid (C24:1) (Fig. 1G). Surprisingly, we also found the odd chain FAs, FA(15:0) and FA(17:0), increased in serum after CL administration. In contrast, we found that LPE(18:2) and LPE (16:0) decreased in serum 30 min after CL administration, (supplemental Fig. S1).

**Fig. 2:**
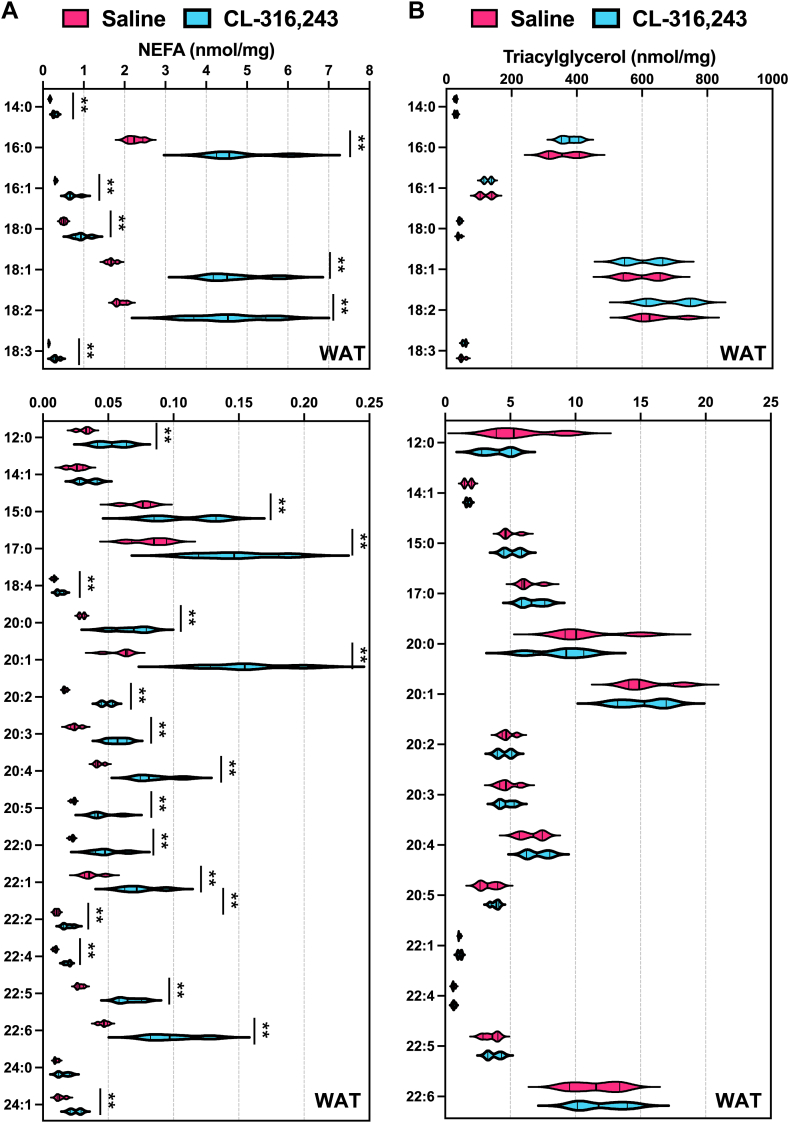
Quantitative analysis of fatty acid composition from WAT after acute activation of adipose tissue lipolysis. A: Nonesterified fatty acids (NEFA) in serum and B: triacylglycerol in inguinal white adipose tissue (WAT) were measured after 30 min of saline or 1 mg/kg CL-316,243 administration (n = 4, ∗: *P* < 0.05, ∗∗: *P* < 0.01).

To test whether the composition of FFAs from the adipose tissue reflected the serum levels, we applied our quantitative analysis to measure FFAs and TAGs in inguinal white adipose tissue (iWAT) after 30 min of CL administration (Fig. 2A). We found that most FFAs increased in response to CL administration around two-fold. The most abundant FFA are FA(16:0), FA(18:1), and FA(18:2) (Fig. 2A). However, TAG levels or composition were not changed after CL treatment (Fig. 2B).

To assess whether other metabolic intermediates changed in serum during the time when FFAs peaked, we measured intermediates of FA oxidation, products of glycolysis, TCA-related substrates, and amino acids by LC-MS (supplemental Fig. S2A–C). We found reduced serum carnitine levels, while glycerol was elevated (supplemental Fig. S2A). Notably, most amino acids decreased in serum, with the exception of cysteine. (supplemental Fig. S2C).

To understand how liver lipids changed, we applied a similar quantitative strategy after 30 min of CL administration. Using a *P* < 0.05 threshold, we found 225 lipids that changed in the liver (supplemental Fig. S2E), with 163 TAG species with varying acyl chains, 11 phosphatidylethanolamine, 13 phosphatidylcholine (PC), 15 phosphatidylglycerol, 3 phosphatidylinositol (PI), 4 phosphotidylserine, 7 fatty acids (FAs), 3 diacylglycerol, 3 lysophosphatidylcholine, 1 lysophosphatidylethanolamine (LPE), 1 sphingomyelin, and 1 phosphatidic acid (PA). Although serum FFAs peaked at 30 min, total hepatic FAs remained unchanged, with the exception of VLCFAs, which were reduced. In contrast, CL-treated mice had a small induction in TAG accumulation in the liver, although not significant (supplemental Fig. S2D). A more detailed analysis showed that there were several TAG species that were elevated with CL administration, with the highest induction in palmitate and linoleate containing TAGs (Table 1).

**Table 1:** Hepatic Lipids 30 mins after CL administration (n = 4)

Tag	Saline (mean)	CL (mean)	*P value*	Fold change
**TG 42:1-FA16:0**	0.0009	0.0020	0.0066	2.33
**TG 42:1-FA18:1**	0.0007	0.0014	0.0014	2.11
**TG 42:2-FA18:2**	0.0009	0.0023	0.0150	2.65
**TG 44:1-FA16:0**	0.0015	0.0039	0.0204	2.55
**TG 44:1-FA18:1**	0.0008	0.0019	0.0043	2.44
**TG 44:2-FA16:0**	0.0020	0.0062	0.0001	3.10
**TG 44:2-FA18:1**	0.0006	0.0014	0.0074	2.39
**TG 44:2-FA18:2**	0.0012	0.0040	0.0007	3.18
**TG 44:3-FA18:2**	0.0007	0.0023	0.0001	3.27
**TG 46:1-FA12:0**	0.0017	0.0048	0.0260	2.75
**TG 46:2-FA12:0**	0.0039	0.0138	0.0010	3.53
**TG 46:2-FA16:0**	0.0035	0.0139	0.0039	3.95
**TG 46:2-FA18:1**	0.0014	0.0040	0.0181	2.80
**TG 46:2-FA18:2**	0.0025	0.0087	0.0077	3.44
**TG 46:3-FA12:0**	0.0009	0.0037	0.0022	4.22
**TG 46:3-FA16:0**	0.0020	0.0068	0.0005	3.41
**TG 46:3-FA16:1**	0.0007	0.0041	0.0129	5.60
**TG 46:3-FA18:1**	0.0012	0.0029	0.0043	2.46
**TG 46:3-FA18:2**	0.0017	0.0071	0.0010	4.28
**TG 46:4-FA18:2**	0.0012	0.0034	0.0008	2.87
**TG 47:2-FA18:2**	0.0011	0.0021	0.0273	2.02
**TG 48:2-FA12:0**	0.0023	0.0046	0.0173	2.01
**TG 48:2-FA14:1**	0.0029	0.0111	0.0134	3.83
**TG 48:3-FA12:0**	0.0033	0.0084	0.0043	2.52
**TG 48:3-FA14:0**	0.0097	0.0344	0.0218	3.54
**TG 48:3-FA14:1**	0.0051	0.0289	0.0019	5.69
**TG 48:3-FA16:0**	0.0061	0.0268	0.0053	4.39
**TG 48:3-FA18:1**	0.0033	0.0113	0.0079	3.48
**TG 48:3-FA18:2**	0.0099	0.0398	0.0097	4.02
**TG 48:3-FA18:3**	0.0016	0.0042	0.0478	2.65
**TG 48:4-FA12:0**	0.0027	0.0069	0.0002	2.58
**TG 48:4-FA14:0**	0.0008	0.0041	0.0029	5.15
**TG 48:4-FA14:1**	0.0010	0.0077	0.0045	7.89
**TG 48:4-FA16:0**	0.0018	0.0060	0.0004	3.39
**TG 48:4-FA16:1**	0.0014	0.0082	0.0172	5.94
**TG 48:4-FA18:1**	0.0013	0.0038	0.0008	2.97
**TG 48:4-FA18:2**	0.0037	0.0144	0.0010	3.85
**TG 48:4-FA18:3**	0.0009	0.0038	0.0122	4.38
**TG 48:5-FA18:2**	0.0012	0.0042	0.0005	3.50
**TG 48:5-FA18:3**	0.0005	0.0021	0.0020	4.51
**TG 49:3-FA15:0**	0.0048	0.0093	0.0094	1.92
**TG 49:3-FA16:0**	0.0025	0.0052	0.0127	2.03
**TG 49:3-FA16:1**	0.0034	0.0082	0.0279	2.45
**TG 49:3-FA18:2**	0.0042	0.0092	0.0153	2.17
**TG 50:2-FA14:1**	0.0004	0.0009	0.0274	2.31
**TG 50:3-FA14:1**	0.0016	0.0070	0.0039	4.50
**TG 50:3-FA16:0**	0.2823	0.6974	0.0400	2.47
**TG 50:3-FA18:0**	0.0009	0.0022	0.0159	2.27
**TG 50:3-FA18:2**	0.2482	0.5814	0.0403	2.34
**TG 50:4-FA14:0**	0.0342	0.0647	0.0428	1.89
**TG 50:4-FA14:1**	0.0029	0.0129	0.0011	4.46
**TG 50:4-FA16:0**	0.0217	0.0603	0.0085	2.79
**TG 50:4-FA16:1**	0.0530	0.1575	0.0490	2.97
**TG 50:4-FA18:1**	0.0066	0.0198	0.0083	3.02
**TG 50:4-FA18:2**	0.0678	0.1524	0.0189	2.25
**TG 50:4-FA18:3**	0.0138	0.0373	0.0361	2.71
**TG 50:4-FA20:4**	0.0007	0.0014	0.0050	2.16
**TG 50:5-FA14:0**	0.0043	0.0122	0.0042	2.85
**TG 50:5-FA16:0**	0.0026	0.0070	0.0008	2.66
**TG 50:5-FA16:1**	0.0045	0.0183	0.0170	4.06
**TG 50:5-FA18:1**	0.0014	0.0043	0.0004	3.09
**TG 50:5-FA18:2**	0.0065	0.0229	0.0014	3.55
**TG 50:5-FA18:3**	0.0057	0.0180	0.0085	3.13
**TG 51:3-FA17:0**	0.0037	0.0079	0.0272	2.14
**TG 51:4-FA16:1**	0.0047	0.0118	0.0305	2.49
**TG 51:4-FA18:2**	0.0264	0.0455	0.0457	1.73
**TG 51:4-FA18:3**	0.0029	0.0059	0.0108	2.03
**TG 51:5-FA18:2**	0.0035	0.0073	0.0097	2.10
**TG 51:5-FA18:3**	0.0022	0.0053	0.0069	2.38
**TG 52:4-FA14:0**	0.0020	0.0034	0.0199	1.72
**TG 52:4-FA16:0**	1.2892	2.1988	0.0256	1.71
**TG 52:4-FA18:0**	0.0021	0.0054	0.0074	2.51
**TG 52:4-FA18:1**	0.4091	0.8184	0.0357	2.00
**TG 52:4-FA18:2**	2.1035	3.6437	0.0247	1.73
**TG 52:4-FA18:3**	0.1653	0.3113	0.0199	1.88
**TG 52:4-FA20:0**	0.0082	0.0151	0.0166	1.83
**TG 52:4-FA20:2**	0.0026	0.0048	0.0194	1.84
**TG 52:4-FA20:4**	0.0085	0.0133	0.0395	1.57
**TG 52:4-FA22:1**	0.0022	0.0044	0.0107	2.03
**TG 52:5-FA14:0**	0.0018	0.0036	0.0174	2.01
**TG 52:5-FA16:0**	0.1572	0.3596	0.0040	2.29
**TG 52:5-FA16:1**	0.1705	0.3959	0.0079	2.32
**TG 52:5-FA18:1**	0.0347	0.0949	0.0051	2.73
**TG 52:5-FA18:2**	0.3469	0.8133	0.0049	2.34
**TG 52:5-FA18:3**	0.1864	0.4282	0.0044	2.30
**TG 52:5-FA20:3**	0.0022	0.0048	0.0227	2.20
**TG 52:5-FA20:4**	0.0084	0.0185	0.0335	2.21
**TG 52:5-FA22:5**	0.0039	0.0090	0.0022	2.31
**TG 52:6-FA14:0**	0.0022	0.0053	0.0039	2.46
**TG 52:6-FA16:0**	0.0115	0.0329	0.0048	2.85
**TG 52:6-FA16:1**	0.0253	0.0834	0.0035	3.29
**TG 52:6-FA18:1**	0.0032	0.0100	0.0015	3.12
**TG 52:6-FA18:2**	0.0314	0.1028	0.0010	3.27
**TG 52:6-FA18:3**	0.0319	0.1056	0.0031	3.32
**TG 52:6-FA20:4**	0.0034	0.0087	0.0028	2.55
**TG 52:6-FA22:6**	0.0035	0.0122	0.0028	3.46
**TG 52:7-FA18:1**	0.0006	0.0014	0.0065	2.17
**TG 52:7-FA20:5**	0.0030	0.0069	0.0280	2.30
**TG 52:7-FA22:6**	0.0018	0.0066	0.0045	3.61
**TG 52:8-FA18:2**	0.0005	0.0017	0.0026	3.55
**TG 53:0-FA16:0**	0.0290	0.0522	0.0464	1.80
**TG 53:4-FA16:0**	0.0221	0.0415	0.0141	1.88
**TG 53:4-FA20:4**	0.0005	0.0009	0.0238	1.63
**TG 53:6-FA20:4**	0.0013	0.0022	0.0327	1.61
**TG 54:0-FA16:0**	0.0037	0.0061	0.0210	1.66
**TG 54:4-FA20:1**	0.0148	0.0268	0.0465	1.81
**TG 54:4-FA20:4**	0.0053	0.0084	0.0305	1.60
**TG 54:5-FA18:3**	0.0713	0.1361	0.0246	1.91
**TG 54:5-FA20:2**	0.0066	0.0123	0.0128	1.85
**TG 54:6-FA16:0**	0.0547	0.0965	0.0254	1.76
**TG 54:6-FA16:1**	0.0113	0.0229	0.0339	2.03
**TG 54:6-FA18:1**	0.1181	0.2623	0.0060	2.22
**TG 54:6-FA18:3**	0.1118	0.2536	0.0059	2.27
**TG 54:6-FA20:3**	0.0088	0.0170	0.0188	1.93
**TG 54:6-FA20:4**	0.1021	0.1755	0.0110	1.72
**TG 54:6-FA22:6**	0.0256	0.0524	0.0351	2.05
**TG 54:7-FA16:1**	0.0116	0.0254	0.0292	2.20
**TG 54:7-FA18:1**	0.0112	0.0286	0.0019	2.55
**TG 54:7-FA18:2**	0.1066	0.2550	0.0072	2.39
**TG 54:7-FA18:3**	0.0615	0.1555	0.0060	2.53
**TG 54:7-FA20:4**	0.0151	0.0333	0.0038	2.21
**TG 54:7-FA22:5**	0.0034	0.0065	0.0343	1.90
**TG 54:8-FA18:2**	0.0109	0.0305	0.0016	2.81
**TG 54:8-FA18:3**	0.0115	0.0371	0.0013	3.23
**TG 54:8-FA20:4**	0.0013	0.0039	0.0006	2.88
**TG 54:8-FA20:5**	0.0155	0.0319	0.0303	2.06
**TG 54:8-FA22:6**	0.0153	0.0310	0.0171	2.03
**TG 55:1-FA18:1**	0.0141	0.0259	0.0231	1.83
**TG 55:2-FA18:2**	0.0181	0.0319	0.0492	1.77
**TG 55:5-FA20:4**	0.0018	0.0028	0.0218	1.54
**TG 56:1-FA16:0**	0.0034	0.0052	0.0146	1.52
**TG 56:1-FA18:1**	0.0043	0.0066	0.0260	1.52
**TG 56:4-FA16:0**	0.0044	0.0059	0.0407	1.36
**TG 56:4-FA20:4**	0.0012	0.0022	0.0005	1.79
**TG 56:6-FA18:3**	0.0060	0.0114	0.0140	1.89
**TG 56:6-FA20:4**	0.0334	0.0513	0.0391	1.54
**TG 56:6-FA22:6**	0.0044	0.0086	0.0340	1.95
**TG 56:7-FA18:0**	0.0018	0.0034	0.0066	1.84
**TG 56:7-FA18:2**	0.0573	0.0960	0.0346	1.68
**TG 56:7-FA18:3**	0.0054	0.0097	0.0127	1.78
**TG 56:7-FA20:3**	0.0138	0.0251	0.0362	1.82
**TG 56:7-FA20:4**	0.0395	0.0720	0.0100	1.82
**TG 56:7-FA20:5**	0.0182	0.0306	0.0365	1.68
**TG 56:8-FA18:1**	0.0162	0.0284	0.0366	1.76
**TG 56:8-FA18:3**	0.0068	0.0126	0.0054	1.84
**TG 56:8-FA20:4**	0.0235	0.0473	0.0144	2.01
**TG 56:8-FA20:5**	0.0350	0.0655	0.0230	1.87
**TG 56:9-FA18:3**	0.0064	0.0134	0.0064	2.08
**TG 56:9-FA20:4**	0.0054	0.0122	0.0065	2.26
**TG 56:9-FA20:5**	0.0226	0.0469	0.0193	2.08
**TG 58:10-FA20:4**	0.0044	0.0080	0.0067	1.81
**TG 58:10-FA20:5**	0.0028	0.0049	0.0237	1.73
**TG 58:10-FA22:5**	0.0041	0.0081	0.0343	1.95
**TG 58:2-FA18:1**	0.0025	0.0035	0.0099	1.39
**TG 58:5-FA18:1**	0.0032	0.0047	0.0371	1.46
**TG 58:6-FA16:0**	0.0027	0.0041	0.0432	1.53
**TG 58:7-FA16:0**	0.0059	0.0104	0.0053	1.78
**TG 58:7-FA18:0**	0.0040	0.0061	0.0391	1.53
**TG 58:7-FA18:2**	0.0067	0.0107	0.0333	1.60
**TG 58:7-FA22:6**	0.0099	0.0166	0.0303	1.67
**TG 58:8-FA20:4**	0.0044	0.0064	0.0245	1.46
**TG 58:9-FA20:4**	0.0036	0.0056	0.0344	1.53
**TG 58:9-FA22:5**	0.0111	0.0205	0.0428	1.84

### Time-dependent changes in serum and liver lipids highlight dynamic lipid remodeling

**Fig. 3:**
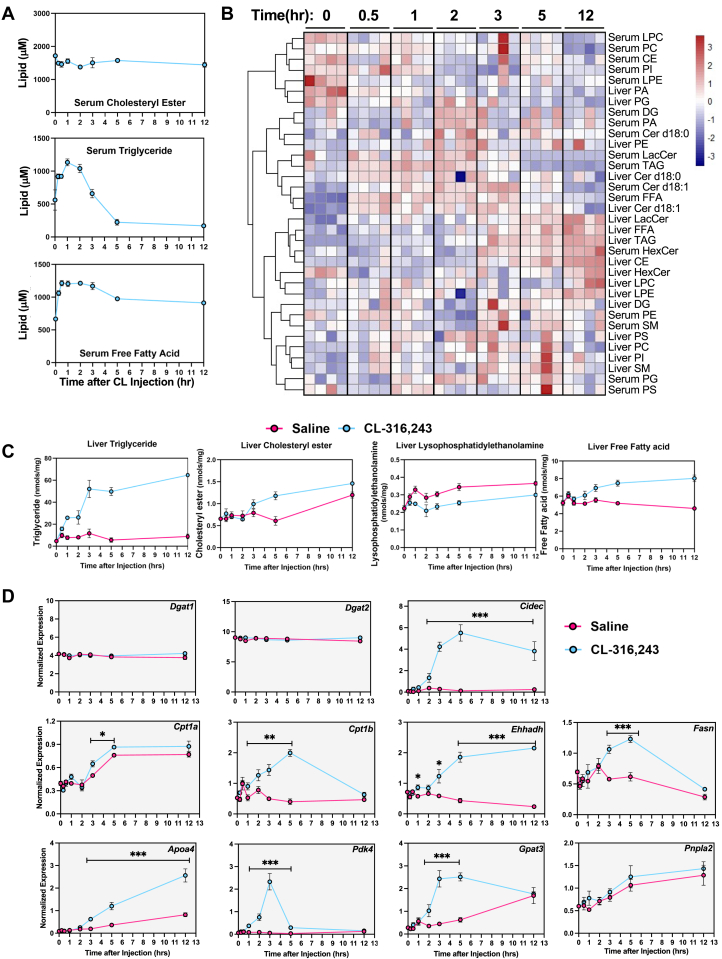
Time-dependent changes in serum and liver lipids after CL-316,243 administration. A: Time-dependent changes in serum cholesteryl ester (CE), triglyceride (TAG), and free fatty acids (FFA) after CL-316,243 administration. (time points 0, 0.5, 1, 2, 3, 5, 12 h). B: Cluster analysis of major lipid classes. (time points 0, 0.5, 1, 2, 3, 5, 12 h). C: Liver triglycerides, CE, lysophosphatidylethanolamine, and FFAs measured after saline or CL-316,243 administration. D: Dynamic changes in hepatic genes expression with activation of adipose tissue lipolysis. Tissues were collected a designated times after a single dose of CL treatment. (n = 4, ∗: *P* < 0.05, ∗∗: *P* < 0.01, ∗∗∗: *P* < 0.005).

A single time point only provided a snapshot of lipid changes; to explore the dynamic changes in serum, we isolated serum from mice at 15 min, 30 min, 1 h, 2 h, 3 h, 5 h, and 12 h after the activation of lipolysis. The data showed serum FAs had been upregulated to the peak concentration in 30 min and sustained at higher levels. In contrast, serum TAG concentration peaked in 1 h. Then the TAG concentration dropped down to a lower level than the initial time point. In addition, we found that CE concentration did not change in the serum (Fig. 3A).

To assess how liver lipids changed during this time, we completed lipidomic analysis by LC-MS for liver samples after a single dose CL injection at various time points (30 min, 1 h, 2 h, 3 h, 5 h, and 12 h). Clustered with the serum and liver lipid species data (supplemental Fig. S3), serum FAs and TAGs were increased earlier than liver FAs and TAGs. Through correlation analysis, we confirmed that some of the other signal lipids, such as CER (both in the serum and liver), were coregulated with serum FAs (Fig. 3B). We found that hepatic TAGs, FAs, and CEs increased in livers (Fig. 3C). Liver TAGs reached the highest concentration at 5 h and remained elevated. In contrast, LPE was reduced in livers after 1 h of CL administration and remained lower throughout the experiment.

### Adipocyte lipolysis promotes the expression of genes involved in lipid handling and hepatic lipid remodeling

With our observation that liver TAGs were elevated with a single dose of CL, we hypothesized that pathways involved in FA oxidation, pyruvate metabolism, and lipid handling would be elevated to accommodate the influx of incoming lipids from adipose tissue. We found that genes involved in regulating pyruvate metabolism, such as Pdk4, were elevated in response to a stimulus of adipocyte lipolysis, suggesting that pyruvate oxidation is blocked in response to influx of lipids from lipolysis. Genes that encode for enzymes involved in FA oxidation, such as Ehhadh (44) and *Cpt1a* and *Cpt1b* (12), TAG synthesis genes *Gpat3 and Fasn* (45, 46), VLDL lipoprotein gene *Apoa4* (12), and lipid droplet biogenesis (*Cidec*), were upregulated after CL administration compared to saline controls. However, *Dgat1*, *Dgat2*, *and Pnpla2* expression did not change with CL administration (Fig. 3D).

**Fig. 4:**
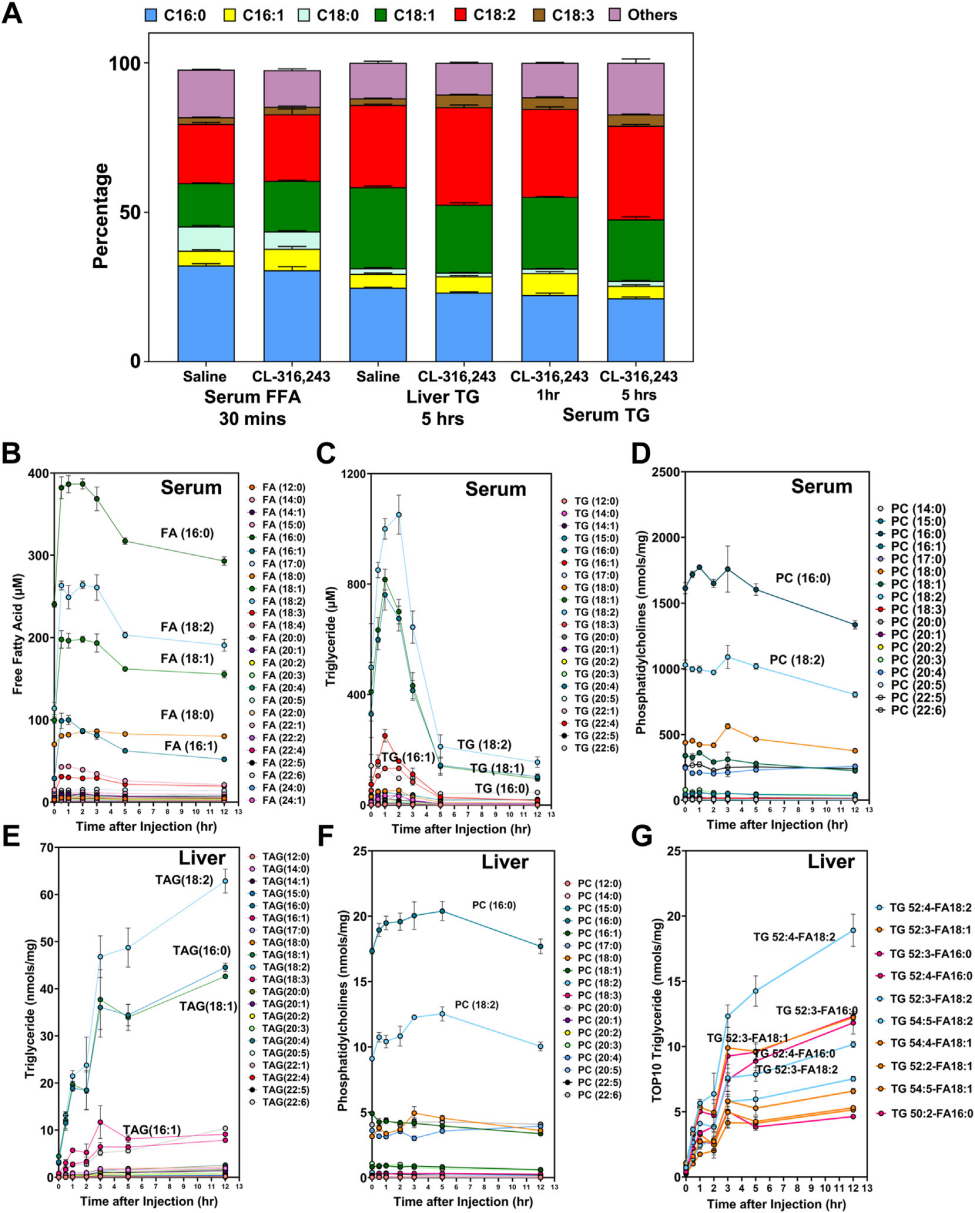
Fatty acid composition of serum and liver triglycerides after CL administration. A: Fatty acid composition of serum FFAs, liver triglycerides, and serum triglycerides as a percentage of total lipid class. B: Time-dependent changes in individual fatty acid species in serum after giving a single dose of 1 mg/kg of CL-316,243. C: Fatty acid composition of serum triglycerides (one of three tails) after giving a single dose of 1 mg/kg CL-316,243. D: Fatty acid composition of serum phosphatidylcholines (one of three tails) after a single dose of 1 mg/kg CL-316,243. E: Fatty acid composition of liver triglycerides (one of three tails) after a single dose of 1 mg/kg of CL-316,243. F: Fatty acid composition of liver phosphatidylcholines after a single dose of 1 mg/kg CL-316,243. G: Fatty acid composition of top10 liver TAG species. Each species has carbon number, number of double bonds, and one of the acyl chains associated with TAG species.

Although serum FFAs peaked after 30 min and were associated with several hepatic lipid species as noted above, hepatic FFA content did not change significant following adipocytes lipolysis. However, the composition of FAs within hepatic TAGs did change significantly. In particular, the percentage of hepatic TAGs containing palmitoleate (16:1) and linoleate (18:2) increased substantially in response to adipocyte lipolysis. In addition, the composition of hepatic TAGs changed from early (1 h) to late (5 h) time points. Notably, the composition of TAGs at 5 h was similar in serum and liver (Fig. 4A). Together, these data indicate that adipocyte lipolysis enriches linoleate (18:2) in hepatic TAG following adipocyte lipolysis (Fig. 4A, C, E). To address the extent to which acyl chains change in serum FFAs and TAGs, we plotted each lipid’s time course. Surprisingly, we found that palmitate (16:0) was the dominant FFA found in mouse serum, whereas linoleate (18:2) was the most abundant esterified FA within serum TAGs (Fig. 4B, E). For serum PC, palmitate acid (16:0) was still the dominant esterified FA (Fig. 4D). For hepatic TAGs, linoleate (18:2) was the most abundant esterified FA. These data indicate that lipid remodeling occurs primarily within TAGs rather than other lipid species with the liver in the context of adipocyte lipolysis (Fig. 4E, F). To further refine the specific TAG species (e.g. combination of three esterified FAs), we plotted the top ten hepatic TAG species (Fig. 4G). The most abundant hepatic TAG species was TAG (52:4)-FA (18:2), which represents TAG (52:4) with at least an FA (18:2) tail.

### Adipocyte lipolysis increases EE and oxidation of both endogenous and dietary-derived lipids

**Fig. 5:**
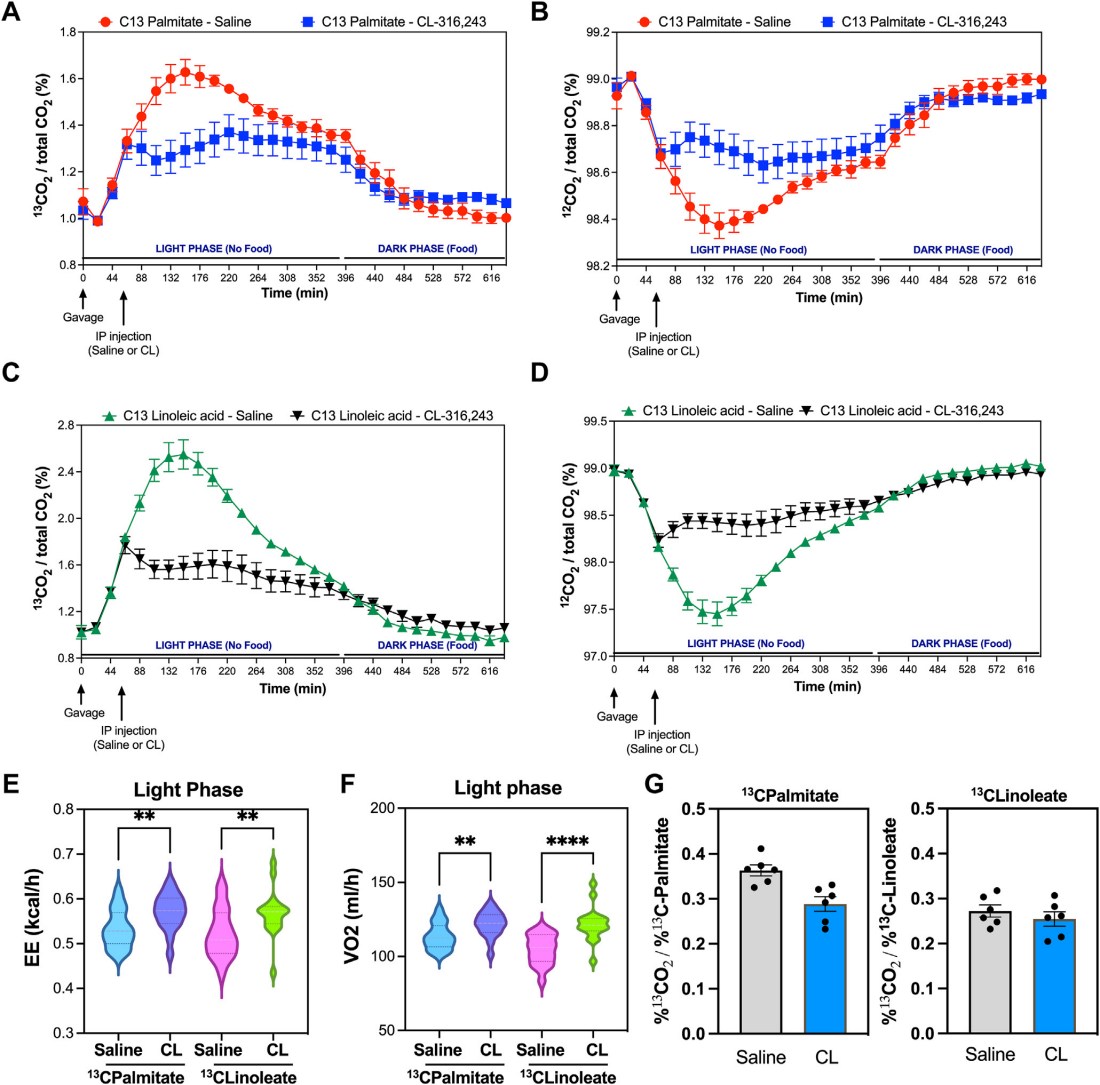
Dietary palmitate and linoleate utilization in response to activation of lipolysis. A: Percentage of ^13^CO_2_ release after gavage with ^13^C-palmitate. B: Percentage of ^12^CO_2_ release after gavage with ^13^C-palmitate. C: Percentage of ^13^CO_2_ release after gavage with ^13^C-linoleic acid. D: Percentage of ^12^CO_2_ release after gavage with ^13^C-linoleic acid. E and F: Area under the Curve (AUC) of Energy Expenditure (EE) (E) or oxygen consumption (F) after saline or CL treatment in mice given ^13^C-palmitate or ^13^C-linoleic acid. G: ^13^C-CO2 production corrected by fractional labeling of serum ^13^C-palmitate or ^13^C-linoleate. Mice were gavaged with U-C13 palmitic acid or U-C13 linoleic acid, followed by intraperitoneal injection an hour later with saline or CL-316,243. Exhaled ^13^CO_2_ for each cage was normalized to total CO2 abundance to quantify changes in systemic palmitic acid or linoleic acid utilization. ∗∗: *P* < 0.01, ∗∗∗∗: *P* < 0.001.

Our observation that TAGs containing linoleate (18:2) are enriched in the hepatic TAG pool suggests that other FAs (e.g. palmitate) may be preferentially hydrolyzed and/or oxidized relative to linoleate (18:2). To test this hypothesis, we measured fat oxidation in mice provided dietary source of U-13C-palmitate or U-13C-linoleate by gavage. We found that with CL treatment, there was significant reduction in products of fat oxidation from U-13C-palmitate and U-13C-linoleate as measured by 13C-CO2 release (Fig. 5A, C). We hypothesized that this resulted from the release of unlabeled FAs from WAT upon activation of lipolysis. To test this hypothesis, we measured the amount of 12C-CO2 release and found that it was indeed elevated (Fig. 5B, D). These findings would suggest that both products of lipolysis and dietary-derived FAs are being utilized during CL treatment. There was no significant difference in RER with CL treatment during the light phase, but CL reduced the RER in the dark phase when food was provided (supplemental Fig. S5A, B). In addition, the CL treatment induced EE and *oxygen consumption (measured by* VO_2_ *test); the data is presented by the* area under the curve (Fig. 5E, F. And the time course data is shown in supplemental Fig. S4C–F. We found that fractional labeling of CO_2_ relative to fractional labeling of palmitate or linoleate was similar in response to CL treatment (Fig. 5F).

### Blocking adipocyte lipolysis prevents hepatic lipid remodeling induced by β3-AR activation

**Fig. 6:**
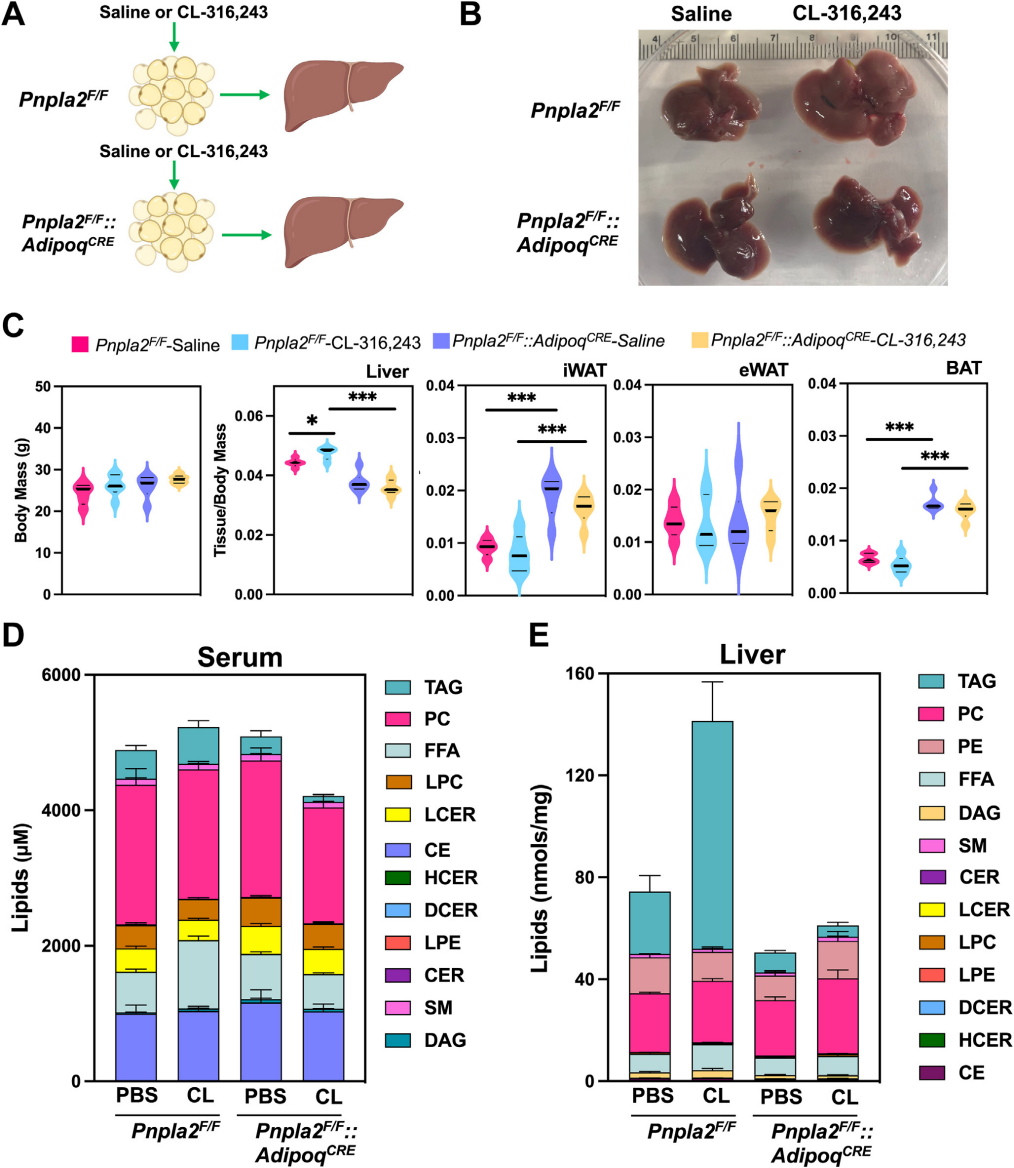
Targeted lipidomic analysis of serum and liver from mice with selective deletion of ATGL in adipocytes. A: Experimental design to test the impact of adipose tissue lipolysis on systemic lipid metabolism and liver. Pnpla2^F/F^ and Pnpla2^F/F^::Adipoq^CRE^ mice were treated with a single dose of saline or 1 mg/kg CL-316,243. B: Morphological changes in livers after saline or CL-316,243 treatment. C: Liver, iWAT, eWAT, and BAT weights corrected for body weight. D and E: Stacked graphs showing serum lipids (D) and liver lipids (E) from major lipid classes. (n = 5–7, ∗: *P* < 0.05, ∗∗∗: *P* < 0.005). ATGL, adipose tissue triglyceride lipase; iWAT, inguinal white adipose tissue; BAT, brown adipose tissue.

**Fig. 7:**
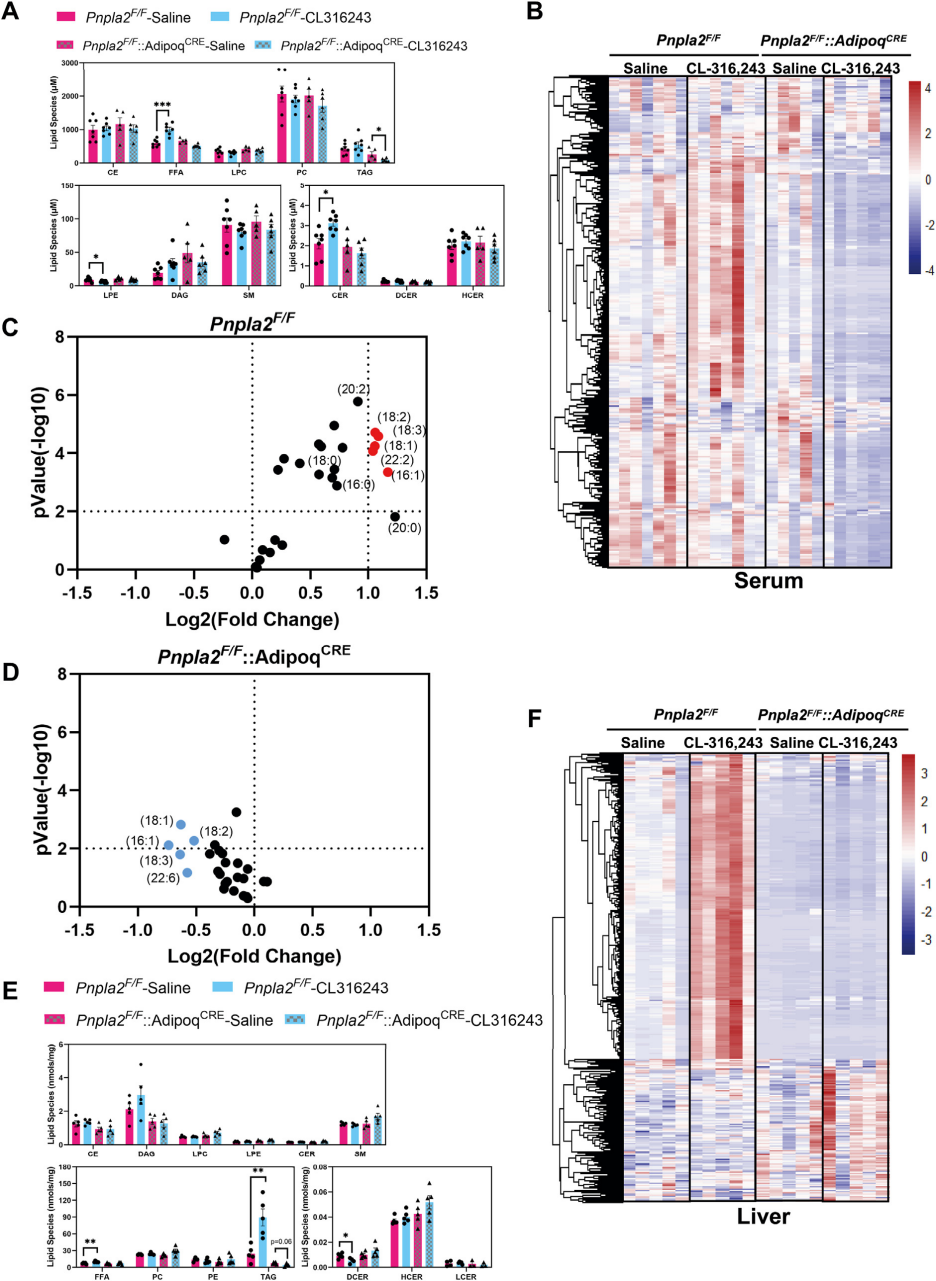
Inhibition of adipose tissue lipolysis prevents lipid remodeling in the liver. A: Major lipid classes in serum after 5 h of saline or CL administration (n = 5–7. B: Heatmap for all four groups in serum lipidome. C: Volcano plot of LC-MS–based FFAs from the plasma of Pnpla2^F/F^ group. FFAs that are increased 2-fold after CL administration and have a *P*-value below 0.01 are labeled in *red*. D: Volcano plot of LC-MS–based FFAs from the plasma of Pnpla2^F F^::Adipoq^CRE^ group. FFAs that are increased 2-fold after CL administration and have a *P*-value below 0.01 are labeled in *red*. E: Major lipid classes in liver after saline or CL administration. (n = 5, ∗: *P* < 0.05, ∗∗: *P* < 0.01, ∗∗∗: *P* < 0.005). E: Heatmap for all four groups in liver lipidome. F: Heat map highlighting liver lipid changes after saline or CL administration.

The ATGL enzyme (*Pnpla2*) catalyzes the first step of TAG hydrolysis, generating DAGs and FFAs (30). To address which lipid changes were directly due to adipocyte lipolysis in response to CL-316,243, we generated mice lacking ATGL in adipocytes (Fig. 6A). Following CL administration (5 h), livers of control *Pnpla2*^*F/F*^ mice developed a pale appearance, but not *Pnpla2*^*F/F*^*::Adipoq*^*CRE*^ mice (Fig. 6B). In addition, control *Pnpla2*^*F/F*^ mice had increased liver/body weight ratio in response to CL-316,243; however, *Pnpla2*^*F/F*^*::Adipoq*^*CRE*^ mice did not have greater liver weight in response to CL-316,243. Loss of ATGL increased the weight of iWAT and BAT, but not epididymal adipose tissue (Fig. 6C). During the acute treatment with CL, we did not find changes in body weight between *Pnpla2*^*F/F*^ and *Pnpla2*^*F/F*^*::Adipoq*^*CRE*^ mice (Fig. 6C). We found that serum FFAs were increased after 5 h of CL administration in the control group (*Pnpla2*^*F/F*^), while *Pnpla2*^*F/F*^*::Adipoq*^*CRE*^ lacked a similar increase (Figs. 6D and 7A). Similarly, lack of ATGL in adipocytes reduced serum TAGs. In contrast, CL administration increased liver TAGs, while *Pnpla2*^*F/F*^*::Adipoq*^*CRE*^ mice were protected (Figs. 6E and 7E).

**Fig. 8:**
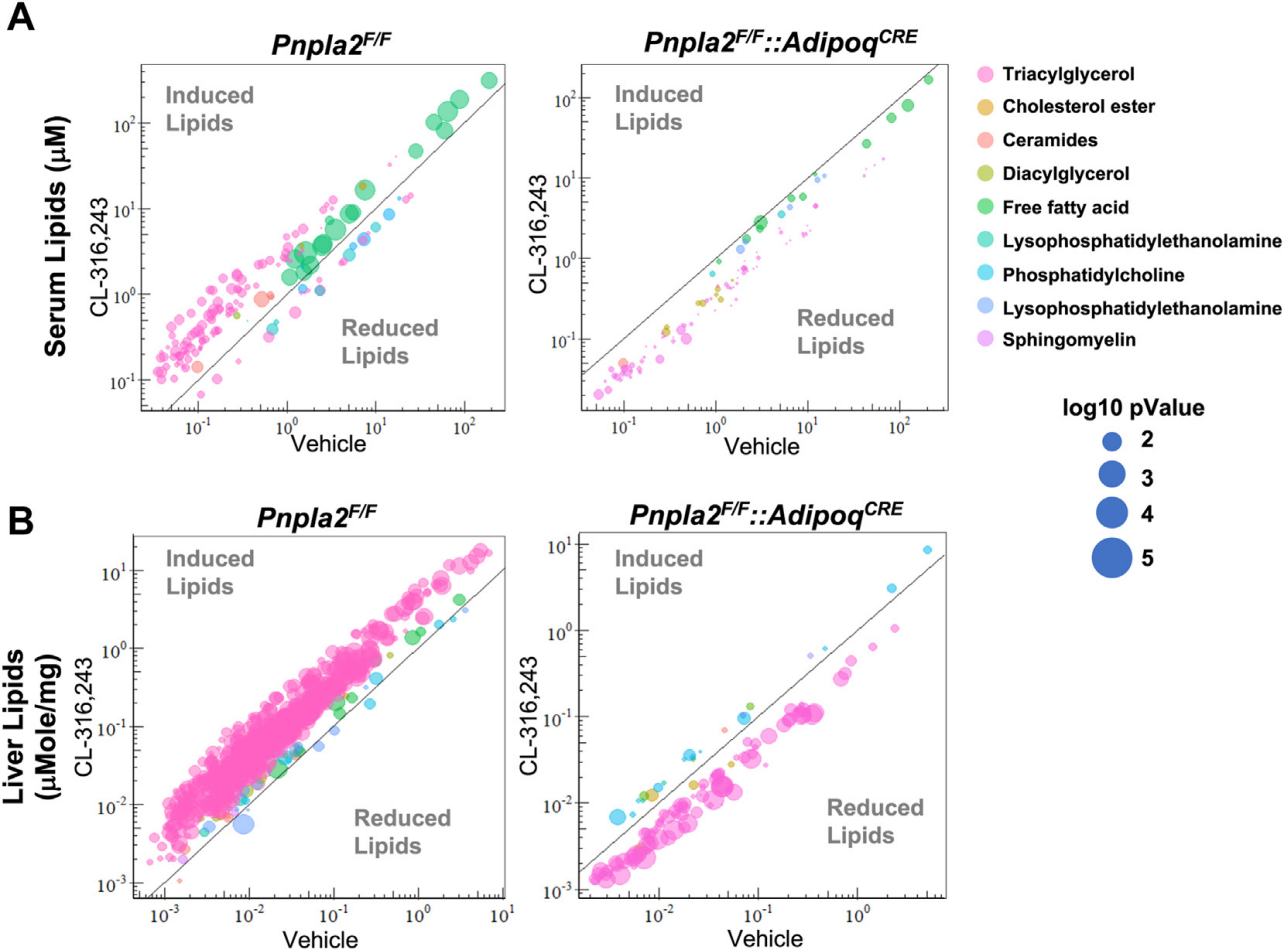
Quantitative changes in lipids from serum and liver in Pnpla2^F/F^ and Pnpla2^F/F^::Adipoq^CRE^ mice. Distribution of significant serum lipids (A) and liver lipids (B) by lipid classes. The Y-axis represents the lipid concentration after CL treatment, and X-axis represents the lipid concentration with vehicle treatment. Dot diameter represents –log10(*P*-value). (n = 5–7).

To understand the impact of adipocyte lipolysis on circulating lipids, we completed lipidomic analysis of the serum after 5 h of CL administration. In the serum, we identified 583 lipids (supplemental Fig. S5A). We found that several long-chain FA species were significantly elevated in the *Pnpla2*^*F/F*^ mice in response to CL; in contrast, *Pnpla2*^*F/F*^*::Adipoq*^*CRE*^ mice had reduced levels of palmitate (16:0), palmitoleate (16:1), oleate (18:1), stearate (18:0), linoleate (18:2), a-linolenic acid(18:3) (Fig. 7C, D). We completed cluster analysis of 583 lipids in *Pnpla2*^*F/F*^ and *Pnpla2*^*F/F*^*::Adipoq*^*CRE*^ mice (Fig. 7B). We found that 143 were significantly changed between saline and CL in control mice; however, *Pnpla2*^*F/F*^*::Adipoq*^*CRE*^ mice had reduced serum levels of several TAG species, FFAs, CERs, and LPEs (Fig. 8A). Compared with the *Pnpla2*^*F/F*^*::Adipoq*^*CRE*^ and *Pnpla2*^*F/F*^ group, 37 significant lipids overlapped. However, these lipids changed in the opposite direction (supplemental Fig. S6B, C). For specific serum lipid concentrations, we plotted that data in supplemental Fig. S6.

To test the impact of adipocyte lipolysis on liver lipids, we completed lipidomic analysis in control and Ko mice (*Pnpla2*). We could quantitatively assess 731 lipids that were identified in the liver (supplemental Fig. S5D). In *Pnpla2*^*F/F*^ controls, we found that CL treatment increased both liver TAGs and FFAs (Fig. 7E). In *Pnpla2*^*F/F*^*::Adipoq*^*CRE*^ mice, CL did not increase liver TAGs (Fig. 7E). We completed cluster analysis based on the 731 lipids from the control group; *Pnpla2*^*F/F*^*::Adipoq*^*CRE*^ did not change for these lipids (Fig. 7F). There are 100 significant lipids overlapped here. However, for lipids species, hepatic TAGs decreased compared with saline treatment (supplemental Fig. S5E, F). For all specific hepatic lipid species, we plotted that data in supplemental Fig. S7.

### Blocking adipocyte lipolysis attenuates TAG accumulation in the liver

To understand how adipocyte lipolysis impacts serum and liver lipids, we plotted changes in lipid classes with a *P*-value < 0.05. We found 117 lipids induced by CL in the *Pnpla2*^*F/F*^ group in serum and no lipids were induced by CL in *Pnpla2*^*F/F*^*::Adipoq*^*CRE*^ group (Fig. 8A). In the liver, we found 507 lipids increased with activation of adipocyte lipolysis in the *Pnpla2*^*F/F*^ group and 22 lipids increased in the *Pnpla2*^*F/F*^*::Adipoq*^*CRE*^ group (Fig. 7F). For the serum samples, in the control group, the most abundant upregulated lipids were FAs and TAGs (Fig. 8). Also, there are a few CERs increased, such as CER(16:0) and CER(24:1) (Fig. 8A). Notably, the combination of blocking adipocyte lipolysis and CL treatment reduced serum and liver FAs and TAGs in *Pnpla2*^*F/F*^*::Adipoq*^*CRE*^ mice (Fig. 8B). Of the 507 lipids induced in the liver, 455 TAG species were upregulated with CL administration. The TAG species had varying acyl chain length and saturation.

### Targeted lipidomic analysis of lipid secretome of differentiated white adipocytes

**Fig. 9:**
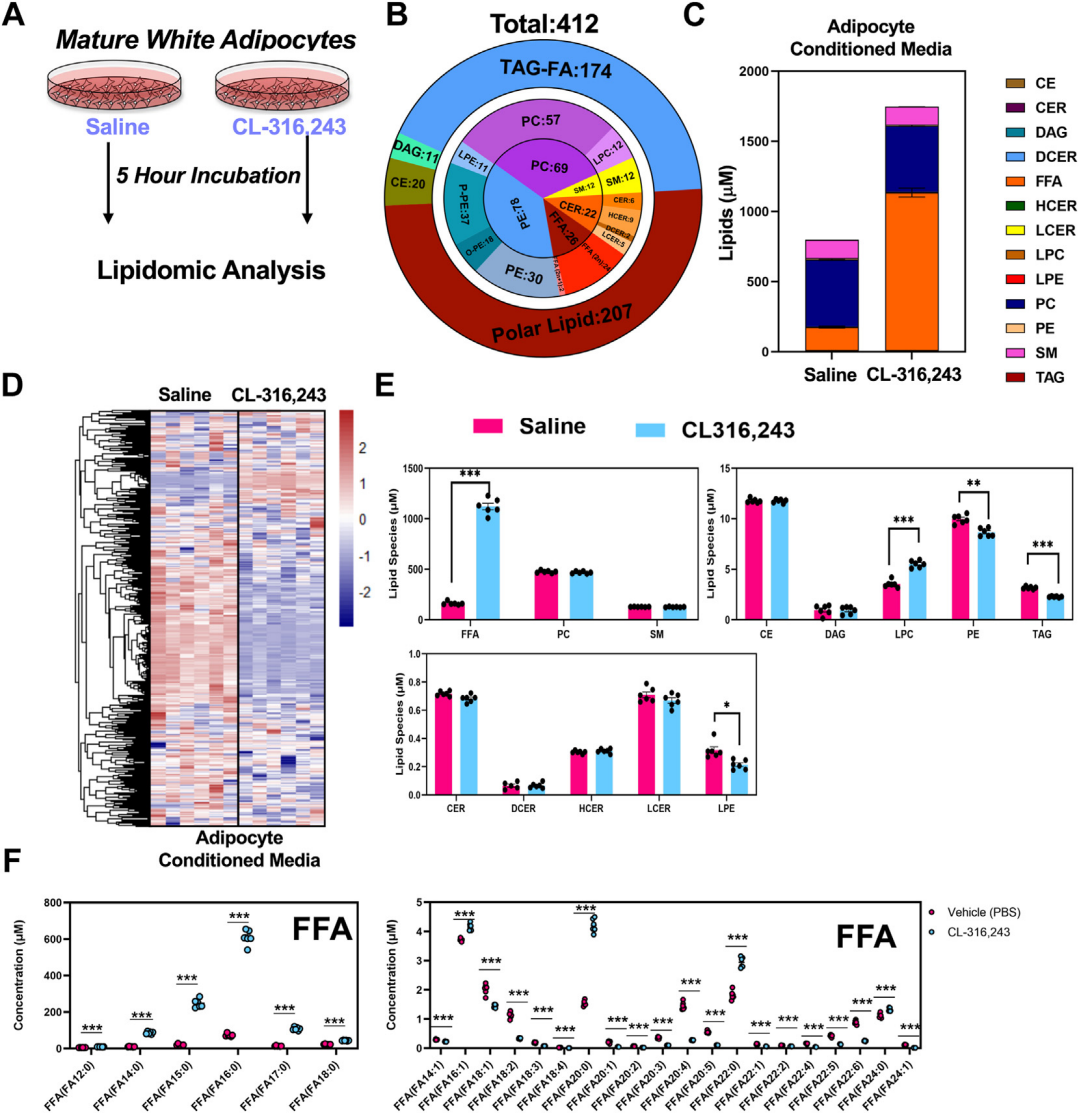
Quantitative lipidomic analysis of conditioned media from differentiated white adipocytes. A: The workflow for condition medium collection and LC-MS analysis. B: Pie graph representing targeted LC-MS analysis. C: The concentration of lipid molecular species of condition medium lipidome grouped by the corresponding lipid class. D: Heatmap displaying all lipid molecular species (targeted LC-MS) between CL and vehicle treatment (n = 6). E: Absolute concentrations of major lipid classes identified in adipocyte conditioned media. F: Fatty acid composition of free fatty acids (FFAs) in media of differentiated adipocytes. (n = 6, ∗: *P* < 0.05, ∗∗: *P* < 0.01, ∗∗∗: *P* < 0.005.

With multiple organs responding to the acute adipocyte lipolysis, it would be hard to analyze the exact lipid secretome from adipose tissue in vivo. We applied an in vitro approach to decipher the composition of the lipids secreted from adipocytes. We differentiated immortalized preadipocytes from iWAT of 12-week-old male mice. We treated cells with vehicle or 10 nM CL for 5 h to stimulate lipolysis and collected the CM to complete untargeted LC-MS analysis (Fig. 9A). Through this analysis we found 559 unique lipid species in the media, of which 125 were increased and 225 decreased with activation of lipolysis (Fig. 9B, C). These included FAs of various lengths. Notably, we identified TAGs in the media, which were decreased with activation of lipolysis.

Using a quantitative lipidomics approach, we identified 412 lipids in CM samples (Fig. 8B, D), 32 increased and 189 decreased (Fig. 9D). FFAs made up the greatest concentration in the CM (Fig. 8E). Notably, we also found LPCs were upregulated by CL treatment. However, PEs, LPEs, and TAGs were downregulated by CL (Fig. 8E). However, it differed with all FAs upregulated in mice serum (except a few VLCFAs, Fig. 1G and supplemental Fig. S5); only saturated FAs increased in the CM (Fig. 9F). The most abundant FA released into the media was palmitate. Further analysis of metabolites showed that glycerol and lactate was increased in the media with CL treatment (supplemental Fig. S8C, D).

Compared with in vivo experiments, we overlapped the lipid species with the CM, 30 min and 5 h serum after CL administration. For all the lipids analyzed, there are 260 lipids overlapped. For significant increased lipids, only FAs (7 FAs, FA(12:0), FA(14:0), FA(16:0), FA(16:1), FA(18:0), FA(20:0), and FA(22:0)) overlapped with the three lipidomic analysis experiments. For the decreased lipids, there were no significant overlapped lipids by three lipidomes. For other specific CM lipids species, we plotted that data in supplemental Fig. S9.

### CM from activation of lipolysis leads to lipid remodeling of hepatocyte lipidome

**Fig. 10:**
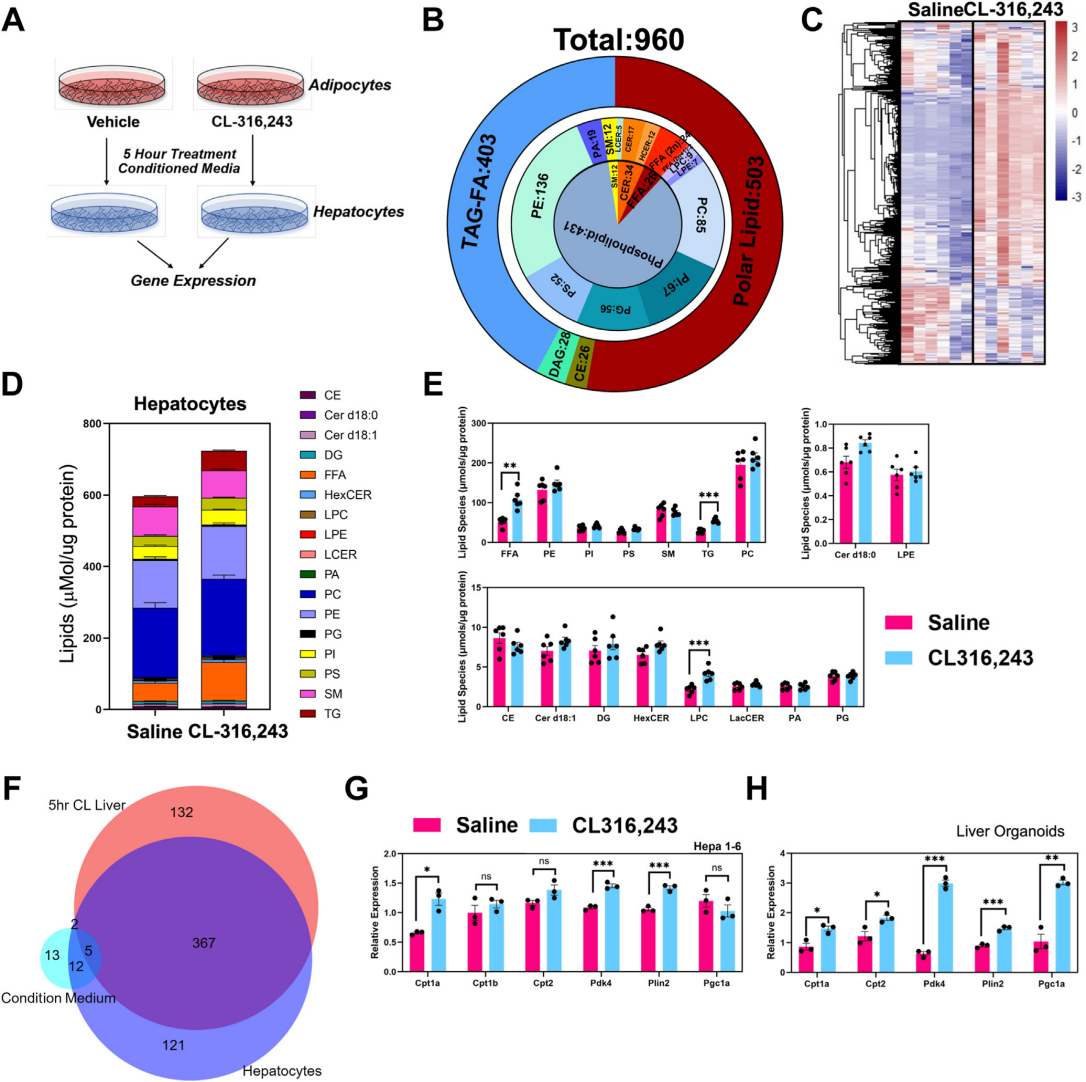
Lipidomic analysis of hepatocytes treated with adipocyte conditioned media. A: The workflow highlighting the production of conditioned media and the treatment of hepatocytes. B: Pie diagram highlights targeted LC-MS analysis of hepatocyte lipidomic analysis grouped by corresponding lipid class. C: Heatmap displays all lipid molecular species comparing hepatocytes treated with conditioned media from adipocytes treated with vehicle or CL-316,243 (n = 6). D: Stacked bar plot with major lipid species comparing hepatocytes treated with adipocyte conditioned media. E: The major lipid classes were grouped by major molecular species. F: Overlapping of characterized lipids species, three Venn graphics present All lipid species overlapping, CL significantly induced lipids overlapping (*P* < 0.05), and CL significantly repressed lipids overlapping (*P* < 0.05). *Red* pie represents liver lipidome after 5 h CL treatment. *Blue* pie represents hepatocyte lipidome after condition medium treatment. Aqua pie represents condition medium lipidome. G: Real-time PCR analysis of HEPA 1–6 cell line in response to condition media from differentiated adipocytes. H: Real-time PCR analysis of mouse liver organoids treated with adipocyte conditioned media. In all experiments, conditioned media was collected from adipocyte after 5 h incubation with saline or CL-316,243.

To test the impact of CM from adipocytes treated with vehicle or CL, we collected CM from mature adipocytes and incubated the media from adipocytes with hepatocytes (HEPA 1–6) for 5 h (Fig. 10A). After removing CM, we completed targeted lipidomic analysis of hepatocytes. We identified 954 lipids in hepatocytes (Fig. 10B, C). The most significant lipids were FAs and TAGs (Fig. 10D, E), which matched with the in vivo experiments (Fig. 4G). Notably, we found sphingomyelin and phospholipids occupied a large part of lipid composition, such as PC, phosphatidylethanolamine, PI, and phosphotidylserine (Fig. 10D). Except for FAs and TAGs, LPCs also increased by CL condition medium (Fig. 10E). For the specific lipids species from CM-treated hepatocytes, we plotted that data in supplemental Fig. S10.

Overlapped with the three lipidomic datasets (CM-treated hepatocytes, 5 h CL liver, and CM), there are a large overlap between the three groups. However, for the statistically significant lipids, CM only have few FAs overlapped with the other two lipidomes, such as FA(14:0), FA(16:0), and FA(16:1). Compared with the lipidomic data from hepatocyte and 5 h liver samples, there are 367 lipids overlapped as CL-induced lipids, including most of saturated, unsaturated FAs, and TAGs (Fig. 9F and supplemental Fig. S7). There were no overlap as decreased lipids (Fig. 10F).

To test whether there is direct communication between adipocytes and hepatocytes, we collected CM from differentiated adipocytes and incubated CM with hepatocytes and liver organoids. We found that CM isolated from CL-treated adipocytes was able to stimulate expression of genes in pathways involved in fat utilization (Fig. 10G, H). The CL condition medium upregulated CPT1a, PDK4, and PLIN2 expression significantly (Fig. 10G). These genes are PPARa and HNF4a targets, and they play critical roles in lipids sensing, particularly for cellular FAs level increasing. To test whether these effects are a result of products of adipocyte lipolysis, we treated cells with ringer’s media that never came into contact with cells. We found that CL did not directly impact gene expression in hepatocytes. In addition, hepatocyte organoids grown in 3D culture using hepatocyte progenitors showed that CM elevated the expression of genes involved in lipids metabolism, including CPT1a, CPT2, PGC1a, and PLIN2. Also, the pyruvate metabolism inhibiting gene PDK4 was induced (Fig. 10H).

## Discussion

NAFLD occurs when excess TAGs accumulate in the liver. The acyl chains in TAGs can originate from various sources, such as dietary intake, de novo FA synthesis, or adipose tissue through the release of FAs during lipolysis. However, the specific contribution of adipose-derived FAs to the hepatic lipid pool is not well understood. Although adipose-derived FAs are thought to contribute to 70% of the TAG pool in human livers (47), there is little known about the direct contribution of exogenous FAs on the liver in vivo. In this study, we set out to develop a quantitative assessment of the time-dependent changes in serum and liver lipids after a single stimulus of adipocyte lipolysis. To our knowledge, there has been no previous in vivo study that has measured the dynamic changes in serum and hepatic lipids upon activation of adipocyte lipolysis. Our findings suggest that the adipose tissue drives profound changes in lipid remodeling in the liver. Interestingly, there are several lipid classes where steady state levels do not change. This comprehensive analysis provides a valuable resource for understanding how dynamic changes in lipid metabolism occur.

WAT is responsible for releasing energy to peripheral tissues via the activation of lipolysis. This process involves the activation of the β3-AR, a G-protein–coupled receptor that signals through PKA to promote release of FFAs from WAT and induce thermogenesis in BAT. A previous study on long-term cold exposure has shown that the lipid composition of serum, WAT, and BAT changes, leading to profound changes in cardiolipin and thermogenesis. Others have highlighted the essential role of adipocyte lipolysis in cold adaptation. However, the effects of acute cold exposure or adipocyte lipolysis on lipid composition are still unclear. Our study aimed to investigate how the acute activation of adipocyte lipolysis impacts hepatic lipid remodeling. Our findings suggest that when adipocyte lipolysis is activated, FFAs increase to 1.5 mM in the serum at 30 min, which we found is primarily composed of palmitate (30.6%), oleate (16.9%), and linoleate (22.3%). During this time, serum TAGs show a transient rise that mirrors the induction of FFAs that seems to be delayed by around 30 min. In contrast, TAG levels in the liver peak at 3 h and are maintained elevated over 12 h during which serum FFA and TG largely revert to normal. These effects are due to CL-mediated activation of white adipocyte lipolysis, as mice lacking ATGL are protected against hepatic TAG accumulation.

As the liver accumulates TAGs, we see an early rise in the expression of genes involved in lipid metabolism and lipid droplet dynamics. Expression of *Gpat3* increased in the liver with activation of adipocyte lipolysis. The induction mirrored the rise in liver TAGs up to 5 h after the initial stimulus. There are four mammalian GPAT enzymes. GPAT1 and GPAT2 are found in the mitochondria, while GPAT3 is localized to the endoplasmic reticulum. The induction of GPAT3 provides one of the key initiation steps in TAG synthesis (48, 49). This rise in GPAT3 likely provides protection against cellular stress caused by the considerable influx of FAs entering from circulation. During the time course, we failed to see changes in the expression of DGAT enzymes, as both *Dgat1* and *Dgat2* expression were unaltered as liver TAGs increased. Although previously it was shown that *Dgat1* expression increases with fasting, a condition where there would be activation of adipocyte lipolysis, we found that WAT lipolysis does not drive the increase in *Dgat1* expression. Perhaps other mechanisms like hormonal regulation of *Dgat1* expression are more relevant in the fasting state. In addition, genes involved in FA oxidation of long-chain FAs like *Cpt1 a/b* and *Ehhadh* were also induced with the activation of adipocyte lipolysis, both reported transcriptional targets of PPARα (50). Another target of PPARα, *Pdk4*, was induced by adipocyte lipolysis. PDK4 phosphorylates pyruvate carboxylase, which results in the inhibition of pyruvate catabolism (51, 52). The inhibition of pyruvate oxidation leads to the preferential use of FAs as a source of energy. Notably, *Cidec*, a regulator of lipid droplet remodeling, is highly induced in the liver and closely follows the changes in liver TAGs (53).

Our analysis suggests that upon activation of adipocyte lipolysis, palmitate (16:0) is the most abundant FFA in the serum and the second and third are linoleate (18:2) and oleate (18:1), respectively. However, quantitative assessment of TAG pool in serum and liver shows that TAGs containing linoleate (18:2) are the most abundant. Linoleic acid (18:2) is an essential polyunsaturated FA that is a precursor for prostaglandins, leukotrienes, and thromboxane (7, 54). Therefore, it’s possible that hepatocytes spare the use of linoleic acid for the synthesis of these potent signaling molecules and store linoleic acid in molecular species like TAGs to prevent their use for FA oxidation. We also considered the possibility that DGAT enzymes could prefer substrate like linoleate to preferentially esterify into the TAG pool. However, DGAT activity assays using an in vitro reconstitution assay where various acyl-CoA species were tested showed a lack of preference for linoleic acid (55). Our isotope tracer studies suggest that activation of WAT lipolysis promotes the use of both dietary-derived FAs and those that are internally stored. In these studies, palmitate or linoleate were provided by gavage, which would be introduced into the blood as chylomicrons, perhaps limiting its use for FA oxidation. Notably, the basal utilization of linoleate was higher than palmitate. Consistent with these findings, isolated mitochondria from rats show higher rates of acylcarnitine production when offered linoleate as the source of FA (56). Alternatively, differences in basal respiration when providing palmitate or linoleate could be explained by the solubility of FAs and their ability to be transported in the intestine. Using an in vivo infusion system to deliver FAs could provide a better comparison of FA utilization.

CL treatment raised liver TAG levels to those seen in mice challenged with high-fat diet (57). To confirm that these effects were due to adipocyte lipolysis, we generated mice lacking ATGL in adipose tissue (*Pnpla2*^*F/F*^*::Adipoq*^*CRE*^). This allowed us to directly test whether lipolysis from WAT was the driver of serum and hepatic lipid changes. We noted that conditional deletion of ATGL in adipocytes completely blocked the CL-induced changes in liver TAGs, highlighting the potential to target adipocyte lipolysis to reverse hepatic steatosis. Notably, CL-treated *Pnpla2*^*F/F*^*::Adipoq*^*CRE*^ mice showed a greater reduction in liver TAGs than saline control, an outcome that is likely due to the activation of BAT thermogenesis (58). Thus, the inhibition of adipocyte lipolysis and activation of thermogenesis has profound effects on liver TAGs and preventing hepatic steatosis.

Our studies suggest that there is direct communication between adipocytes and hepatocytes. In our study, we used an in vitro system to understand how adipocyte-derived lipids impact hepatocyte lipids. In addition to even chain FAs, like FA(14:0), FA(16:0), FA(16:1), and FA(18:0), FA(18:1), FA(18:2), FA(18:3), we identified odd chain FAs in the serum and CM, including FA(15:0) and FA(17:0). Normally, straight odd chain FAs are only synthesized in plants and only exist at a low level in mammals (59). However, in culture, the relative levels of odd-chain FAs were proportionally higher than even-chain FAs. We suspect that FA(15:0) and FA(17:0) are derived from branched-chain FAs through branch chain amino acid metabolism (60). These studies suggest that de novo FA synthesis in vitro primarily drives the synthesis of odd chain FAs. Thus, the composition of FAs in vitro in adipocytes may not reflect the composition in vivo. In addition, the composition of products of lipolysis in adipocytes may vary depending on the source of FBS used for experiments.

We noted significant changes in other lipid classes and we found that LPE is consistently downregulated in vitro and in vivo after CL administration. LPE is a rare phospholipid found in cell membranes. LPE also plays a role in cell-cell signaling and enzyme activation, such as MAPK pathway and *calcium channel activation* (61, 62). The reduction of serum LPE might be a potential mechanism to balance calcium levels in different organs in responding to adipocyte lipolysis. With a reduction of LPE in serum, the activity of calcium channels might be reduced by lower phosphorylation of MAPK. CERs also showed dynamic changes with activation of adipocyte lipolysis. Liver CERs (Cer d18:0, Cer d18:1) and serum CERs (Cer d18:0, Cer d18:1) both increase acutely with the rise in serum FFAs, while liver and serum HexCer cluster together with liver FFA and liver TG. These observations would suggest that CER accumulation is likely driven from excess adipocyte lipolysis; perhaps a compensatory mechanism to deal with FA overload. CERs are linked with nonalcoholic steatohepatitis (63, 64) and insulin resistance (65), conditions where one might expect to see nutrient overload (66). Thus, adipocyte lipolysis is sufficient in driving induction of both serum and liver CERs.

In summary, we provide a detailed analysis of both the serum and liver lipidome in response to activation of adipocyte lipolysis. Here, we demonstrated that the lipids from adipose tissue could remodel the hepatic lipids directly and indirectly. Acute activation of lipolysis led to a dramatic rise in serum lipids, most of which were FAs and CER species. These studies provide a framework to understand the lipid signaling that drives regulation of lipid metabolism in the liver and the genes involved in lipid remodeling.

## Data availability

Data will be shared upon written request to Claudio Villanueva at UCLA through email (cvillanueva@ucla.edu).

## Supplemental data

This article contains supplemental data.

## Conflict of interest

The authors declare that they have no conflicts of interest with the contents of this article.

## Supplemental Data

Supplemental figure 1 The concentration of specific lipid species in serum after 30 minutes of vehicle or CL-316,243 administration. A–J. The concentration of targeted lipid species in serum.

Supplemental figure 2 Serum and liver metabolites after 30 minutes of (CL) CL-316,243 administration. A-C. Serum metabolites after 30 minutes of CL administration. A. Serum carnitine, ac-carnitine, choline, 3-PG (3-phosphoglhycerate), glycerol, G-3-P (Glycerol-3-phosphate), and p-choline (phosphotidylcholine) 30 minutes after saline or CL-316,243 administration. B. Serum Lac (Lactose), Mal (malate), aconitate, citrulline, and succ (succinate) 30 minutes after saline or CL-316,243 administration. C. Serum amino acids after 30 minutes of saline or CL-316243 administration. D. Liver lipid classes change in the liver after 30 minutes of CL administration. E. Cluster analysis of liver lipids comparing saline and CL-316,243 administration, *P* < 0.05. (n = 4, ∗: *P* < 0.05, ∗∗: *P* < 0.01, ∗∗∗: *P* < 0.005).

Supplemental figure 3 Time Course for serum and liver after CL Administration. Cluster analysis showing a heatmap from mouse serum lipids (A) and liver (B) after CL administration.

Supplemental figure 4 Dietary palmitate and linoleate utilization in response to activation of lipolysis. A. B. The respiratory exchange ratio (RER) (VCO2/VO2) during the course of the experiment. Mice were treated with saline or Cl-316,243. C. D. Total Energy Expenditure (EE) with saline or CL-316,243 treatment. E. F. Oxygen consumption with saline or CL-316,243 administration. Mice were treated with 1 mg/kg CL-316,243. Mice were gavaged with ^13^C-palmitate or ^13^C-linoleate after 1 h.

Supplemental figure 5 Comparative analysis of serum lipids from Pnpla2^F/F^ mice and Pnpla2^F/F^::Adipoq^CRE^ mice. A. Pie graph showing serum lipids grouped by the corresponding lipid classes. 583 serum lipids were identified. B. Overlapped Significant CL-induced and CL-reduced serum lipids between between Pnpla2^F/F^ mice and Pnpla2^F/F^::Adipoq^CRE^ mice. Correlation cluster of 37 overlapped significant lipids. C. Heatmap for all four groups in overlapped serum lipids. D. Pie graphic represents mice liver lipid molecular species grouped by the corresponding lipid classes. Totally dentified 583 lipids in serum lipids. E. Overlapped Significant CL-induced and CL-reduced serum lipids between Pnpla2^F/F^ mice and Pnpla2^F/F^::Adipoq^CRE^ mice. F. Heatmap for all four groups in overlapped hepatic lipids.

Supplemental figure 6 Quantitative analysis of serum lipids in Pnpla2^F/F^ and Pnpla2^F/F^::Adipoq^CRE^ mice after 5 h CL administration. Mice were administered a single dose of Saline or 1mg/kg CL-316,243 and sacrificed after 5 hours (n = 5–7).

Supplemental figure 7 Quantitative analysis of liver lipids in Pnpla2^F/F^ and Pnpla2^F/F^::Adipoq^CRE^ mice after 5 h CL administration. Mice were administered a single dose of Saline or 1mg/kg CL-316,243 and sacrificed after 5 hours (n = 5–7).

Supplemental figure 8 Metabolite analysis of condition media from differentiated adipocytes. A. Pie graph of untargeted LC-MS analysis of adipocyte secreted lipids grouped by lipid categories. B. Heatmap displays significantly regulated lipid molecular species (untargeted LC-MS) between CL and vehicle treatment (n = 6, *P* < 0.05). C. Polar metabolites in media. Carnitine, ac-carnitine, choline, 3-PG, glycerol, G-3-P, and p-choline. D. Glycolytic and TCA-cycle intermediates, including citrate, lactate, malate, aconitate, and succinate. E. Measurement of amino acid species in condition media. (n = 5-6, ∗: *P* <0.05, ∗∗: *P* < 0.01, ∗∗∗: *P* < 0.005).

Supplemental figure 9 Quantitative lipid analysis of condition medium of differentiated adipocytes after vehicle or CL-316,243 administration. (n = 6, ∗: *P* < 0.05, ∗∗: *P* < 0.01, ∗∗∗: *P* < 0.005).

Supplemental figure 10 Quantitative lipid analysis of hepatocytes treated with adipocyte conditioned media. Adipocytes were treated with saline or CL-316,243 in. (n = 6, ∗: *P* < 0.05, ∗∗: *P* < 0.01, ∗∗∗: *P* < 0.005).

Cover Art Surface of the adipose tissue using light microscopy. The image of the adipose tissue reflects the mass spec analysis of products of adipose tissue lipolysis highlighted in Zhang et al. Image was captured by Julia Mack and tissue was processed by Mirian Krystel de Siqueira.
