# Supplementary material for: Acute activation of adipocyte lipolysis reveals dynamic lipid remodeling of the hepatic lipidome
Source: J Lipid Res. 2023 Aug 26;65(2):100434. doi: 10.1016/j.jlr.2023.100434 (PMC10839691; doi:10.1016/j.jlr.2023.100434)

Supplemental Figure 1. The concentration of specific lipid species in serum after 30 minutes of CL administration.

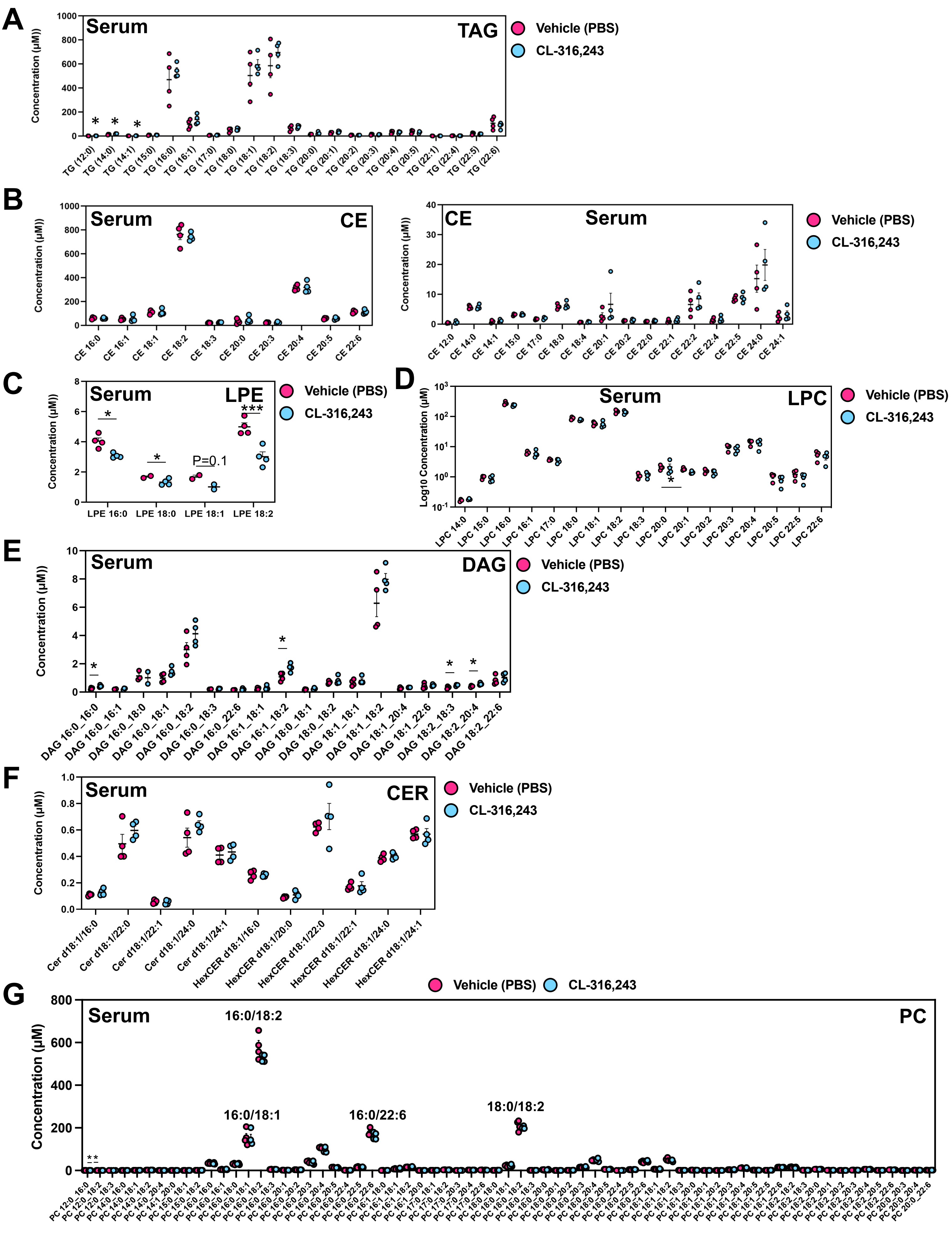

Supplement Figure 1. The concentration of specific lipid species in serum after 30 mins of CL administration.

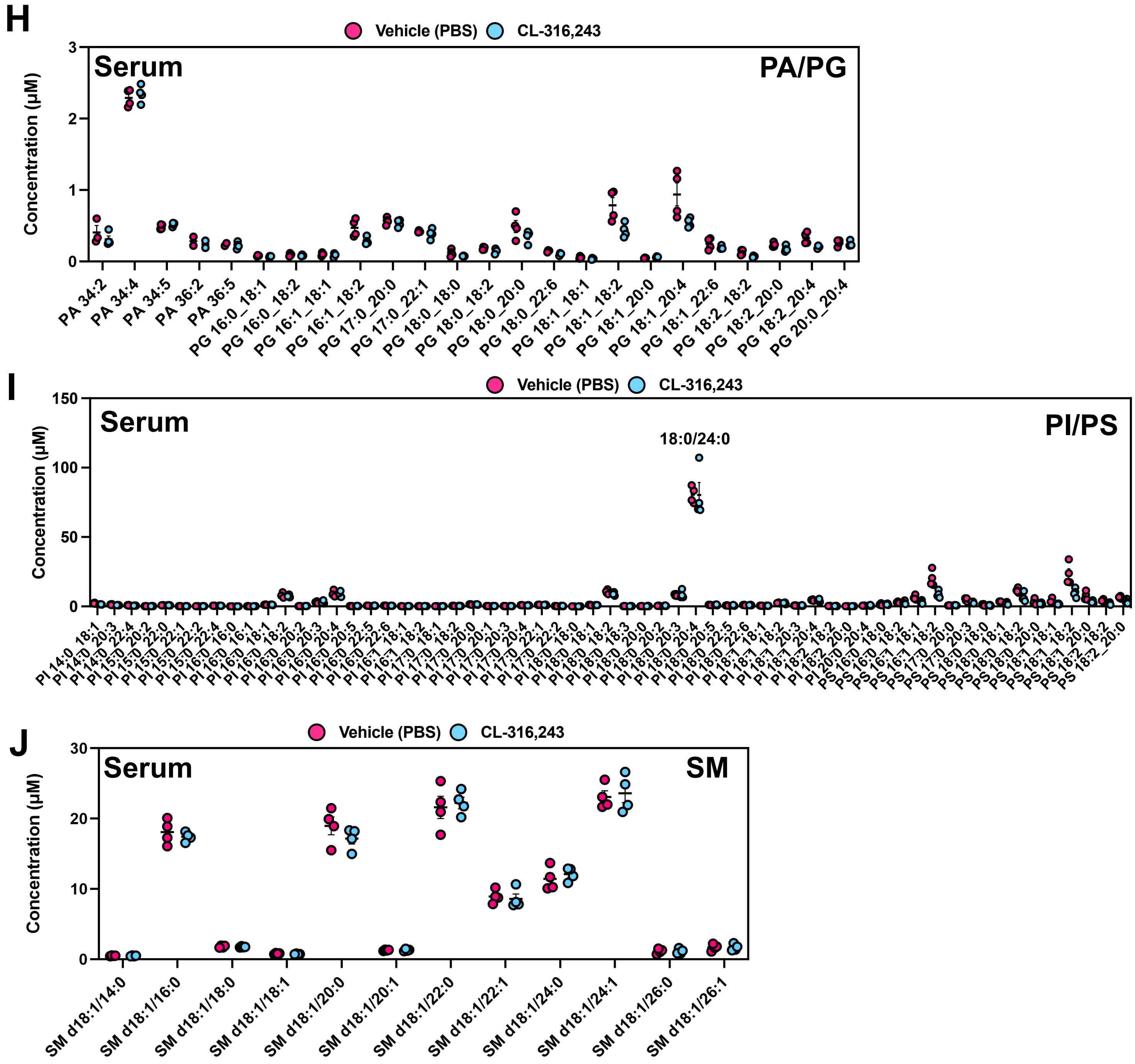

Supplement: Supplemental figure 1 — The concentration of specific lipid species in serum after 30 minutes of vehicle or CL-316,243 administration. A–J. The concentration of targeted lipid species in serum. [file mmc1.pdf]
