# Supplementary figures and images for: Acute activation of adipocyte lipolysis reveals dynamic lipid remodeling of the hepatic lipidome
Source: J Lipid Res. 2023 Aug 26;65(2):100434. doi: 10.1016/j.jlr.2023.100434 (PMC10839691; doi:10.1016/j.jlr.2023.100434)

Supplement Figure 2. Serum and liver metabolites after 30 minutes of CL-316,243 administration.

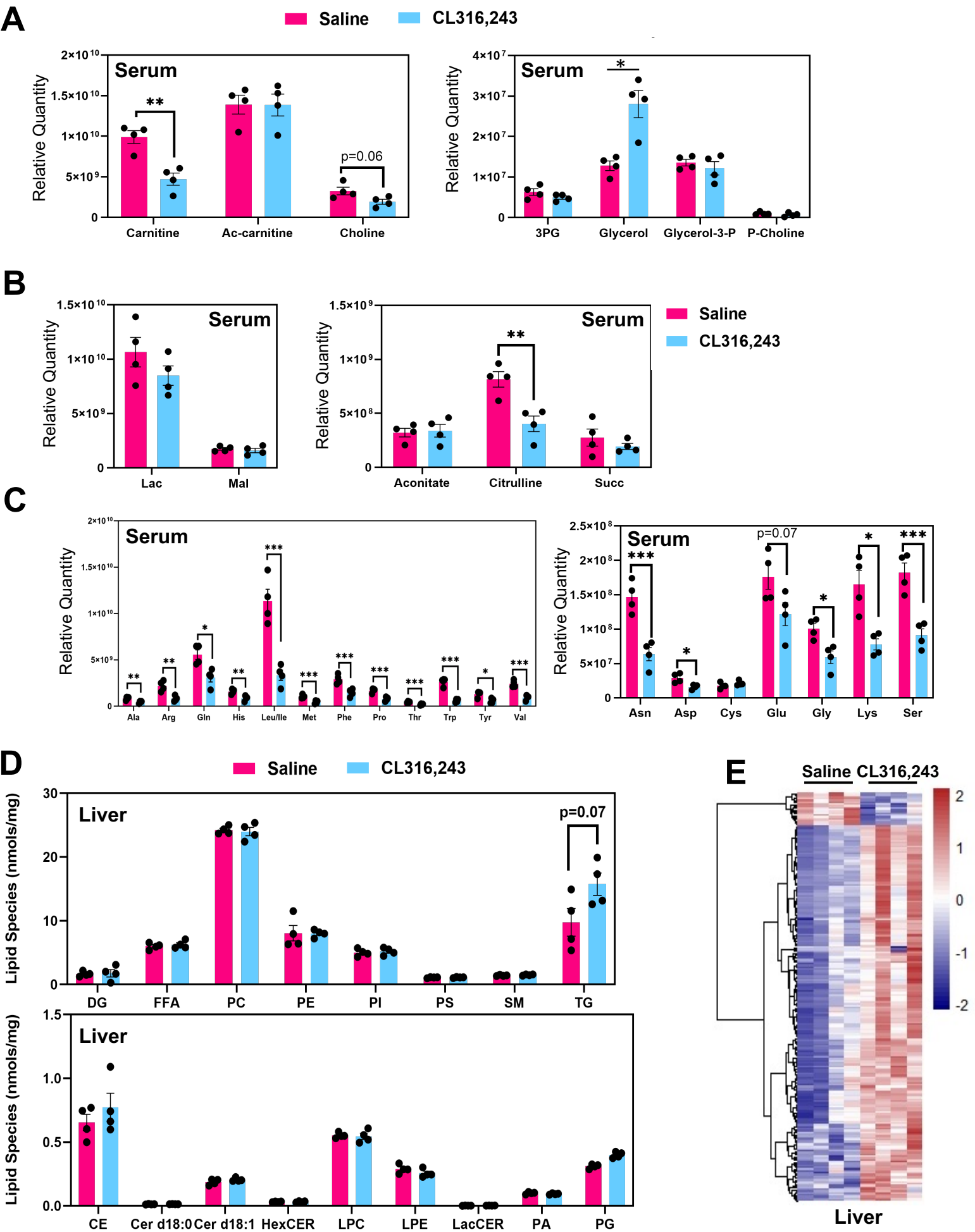

Supplement: Supplemental figure 2 — Serum and liver metabolites after 30 minutes of (CL) CL-316,243 administration. A-C. Serum metabolites after 30 minutes of CL administration. A. Serum carnitine, ac-carnitine, choline, 3-PG (3-phosphoglhycerate), glycerol, G-3-P (Glycerol-3-phosphate), and p-choline (phosphotidylcholine) 30 minutes after saline or CL-316,243 administration. B. Serum Lac (Lactose), Mal (malate), aconitate, citrulline, and succ (succinate) 30 minutes after saline or CL-316,243 administration. C. Serum amino acids after 30 minutes of saline or CL-316243 administration. D. Liver lipid classes change in the liver after 30 minutes of CL administration. E. Cluster analysis of liver lipids comparing saline and CL-316,243 administration, P < 0.05. (n = 4, ∗: P < 0.05, ∗∗: P < 0.01, ∗∗∗: P < 0.005). [file mmc2.pdf]

Supplement Figure 8. Untargeted lipidomic analysis and Condition medium metabolites.

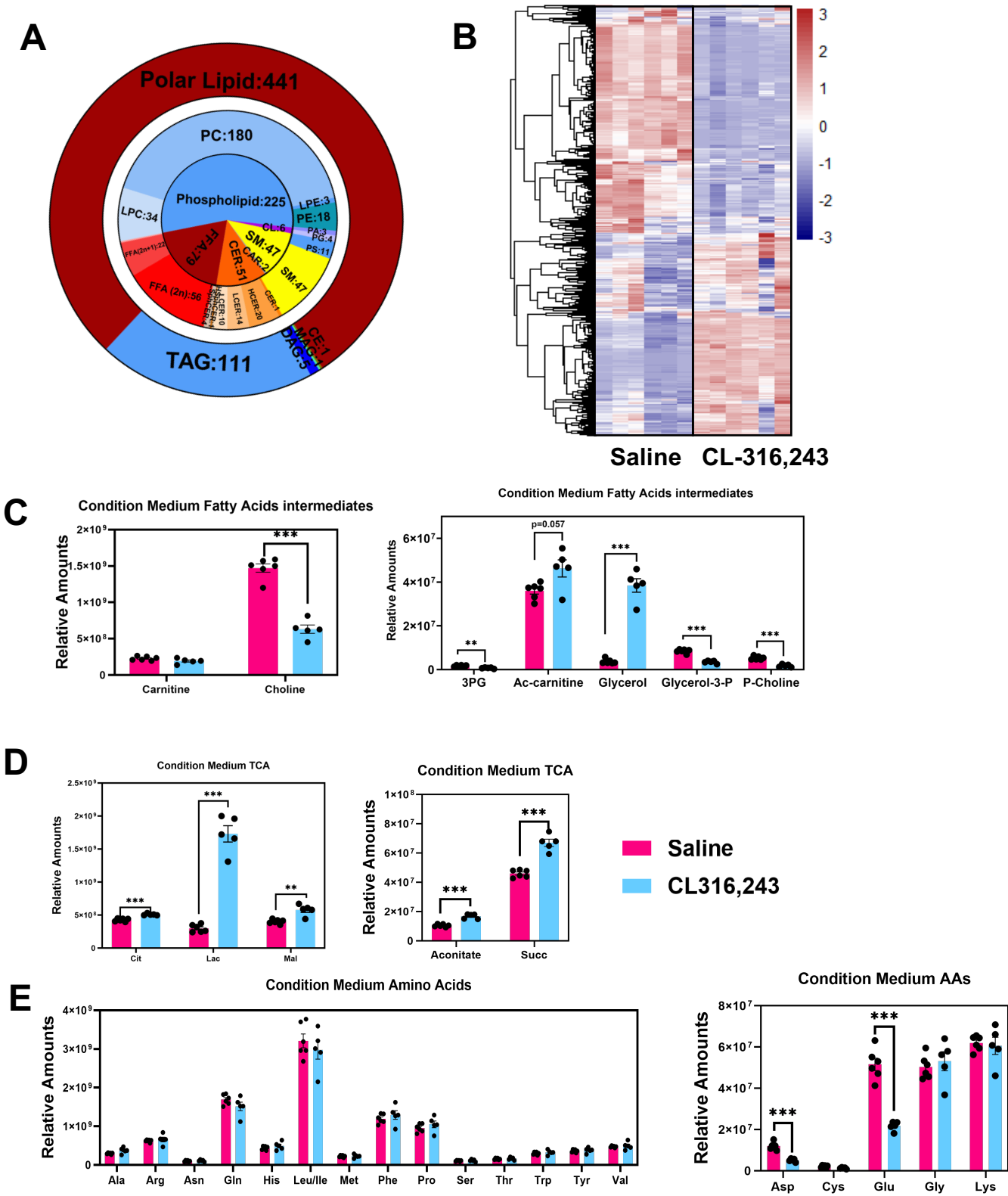

Supplement: Supplemental figure 8 — Metabolite analysis of condition media from differentiated adipocytes. A. Pie graph of untargeted LC-MS analysis of adipocyte secreted lipids grouped by lipid categories. B. Heatmap displays significantly regulated lipid molecular species (untargeted LC-MS) between CL and vehicle treatment (n = 6, P < 0.05). C. Polar metabolites in media. Carnitine, ac-carnitine, choline, 3-PG, glycerol, G-3-P, and p-choline. D. Glycolytic and TCA-cycle intermediates, including citrate, lactate, malate, aconitate, and succinate. E. Measurement of amino acid species in condition media. (n = 5-6, ∗: P <0.05, ∗∗: P < 0.01, ∗∗∗: P < 0.005). [file mmc8.pdf]

**A**

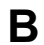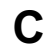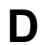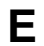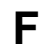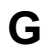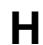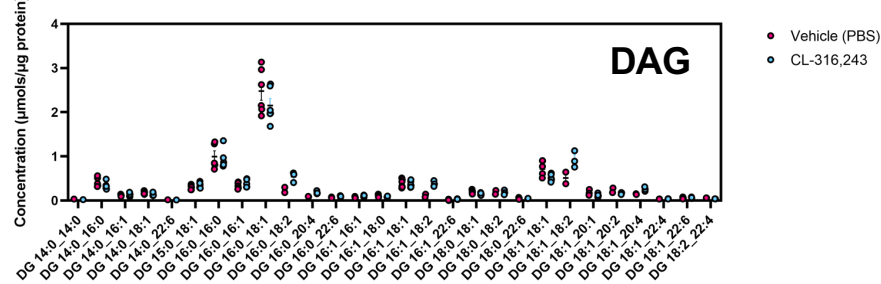

# M

Supplement: Supplemental figure 10 — Quantitative lipid analysis of hepatocytes treated with adipocyte conditioned media. Adipocytes were treated with saline or CL-316,243 in. (n = 6, ∗: P < 0.05, ∗∗: P < 0.01, ∗∗∗: P < 0.005). [file mmc10.pdf]
