# Supplementary material for: Acute activation of adipocyte lipolysis reveals dynamic lipid remodeling of the hepatic lipidome
Source: J Lipid Res. 2023 Aug 26;65(2):100434. doi: 10.1016/j.jlr.2023.100434 (PMC10839691; doi:10.1016/j.jlr.2023.100434)

Supplement Figure 3. Time Course for Serum and liver after CL Administration.

A **Serum Lipids**

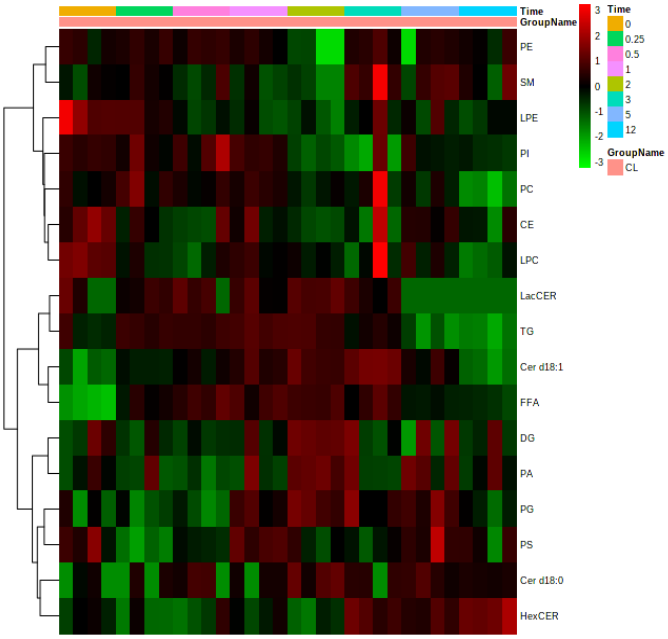

B **Liver Lipids**

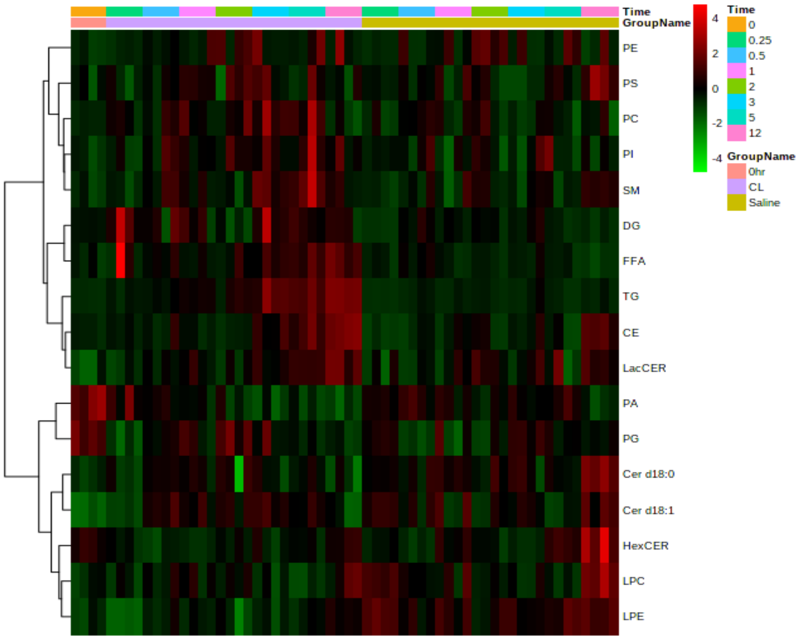

Supplement: Supplemental figure 3 — Time Course for serum and liver after CL Administration. Cluster analysis showing a heatmap from mouse serum lipids (A) and liver (B) after CL administration. [file mmc3.pdf]
