# Supplementary material for: Acute activation of adipocyte lipolysis reveals dynamic lipid remodeling of the hepatic lipidome
Source: J Lipid Res. 2023 Aug 26;65(2):100434. doi: 10.1016/j.jlr.2023.100434 (PMC10839691; doi:10.1016/j.jlr.2023.100434)

Supplement Figure 4. **Dietary palmitate and linoleate utilization in response to activation of lipolysis.**

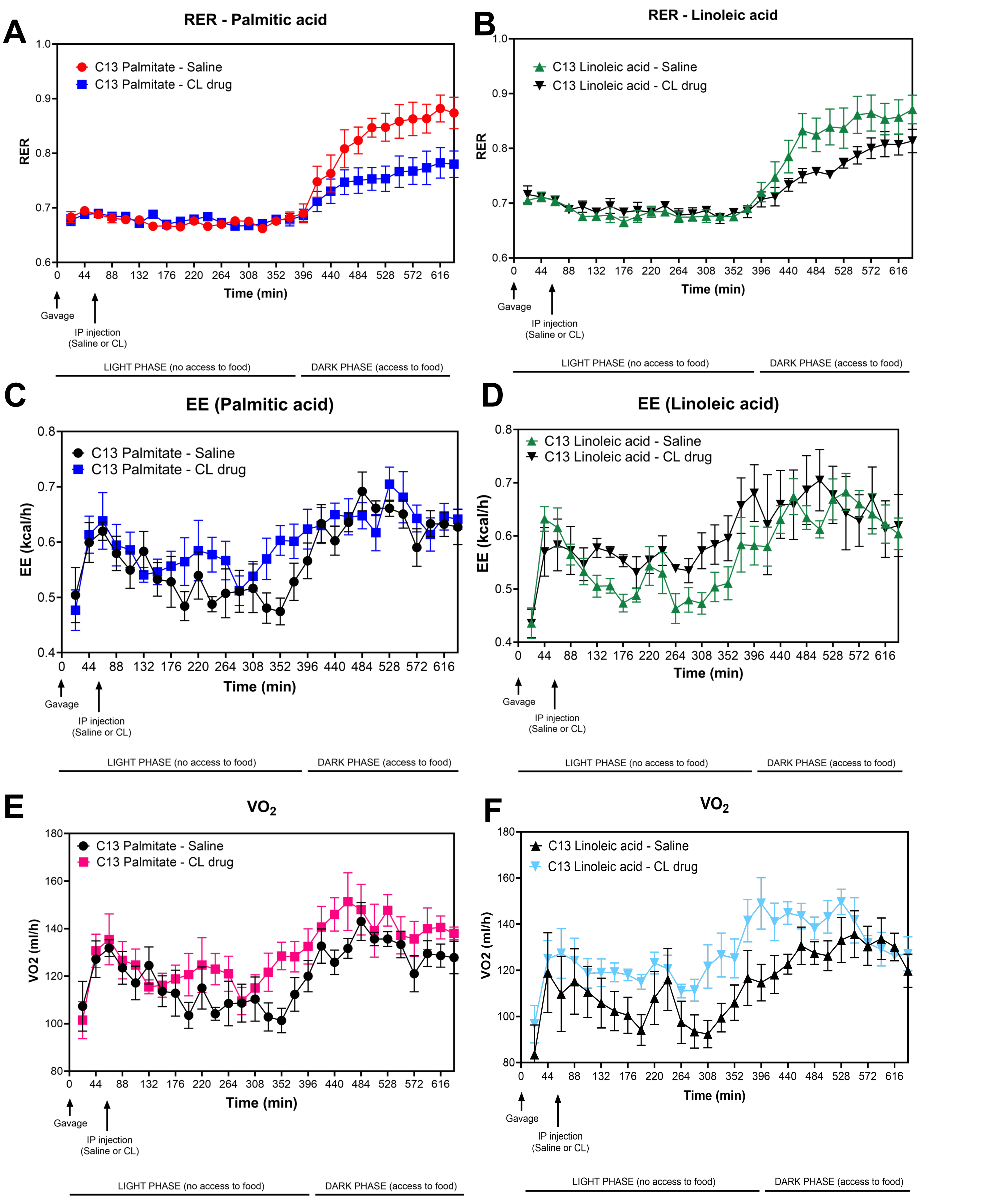

Supplement: Supplemental figure 4 — Dietary palmitate and linoleate utilization in response to activation of lipolysis. A. B. The respiratory exchange ratio (RER) (VCO2/VO2) during the course of the experiment. Mice were treated with saline or Cl-316,243. C. D. Total Energy Expenditure (EE) with saline or CL-316,243 treatment. E. F. Oxygen consumption with saline or CL-316,243 administration. Mice were treated with 1 mg/kg CL-316,243. Mice were gavaged with 13C-palmitate or 13C-linoleate after 1 h. [file mmc4.pdf]
