# Supplementary material for: Acute activation of adipocyte lipolysis reveals dynamic lipid remodeling of the hepatic lipidome
Source: J Lipid Res. 2023 Aug 26;65(2):100434. doi: 10.1016/j.jlr.2023.100434 (PMC10839691; doi:10.1016/j.jlr.2023.100434)

Supplement Figure 5. Significantly changed lipids, Overlapped between *Pnpla2$^{F/F}$* mice and *Pnpla2$^{F/F}$::Adipoq^{CRE}* mice.

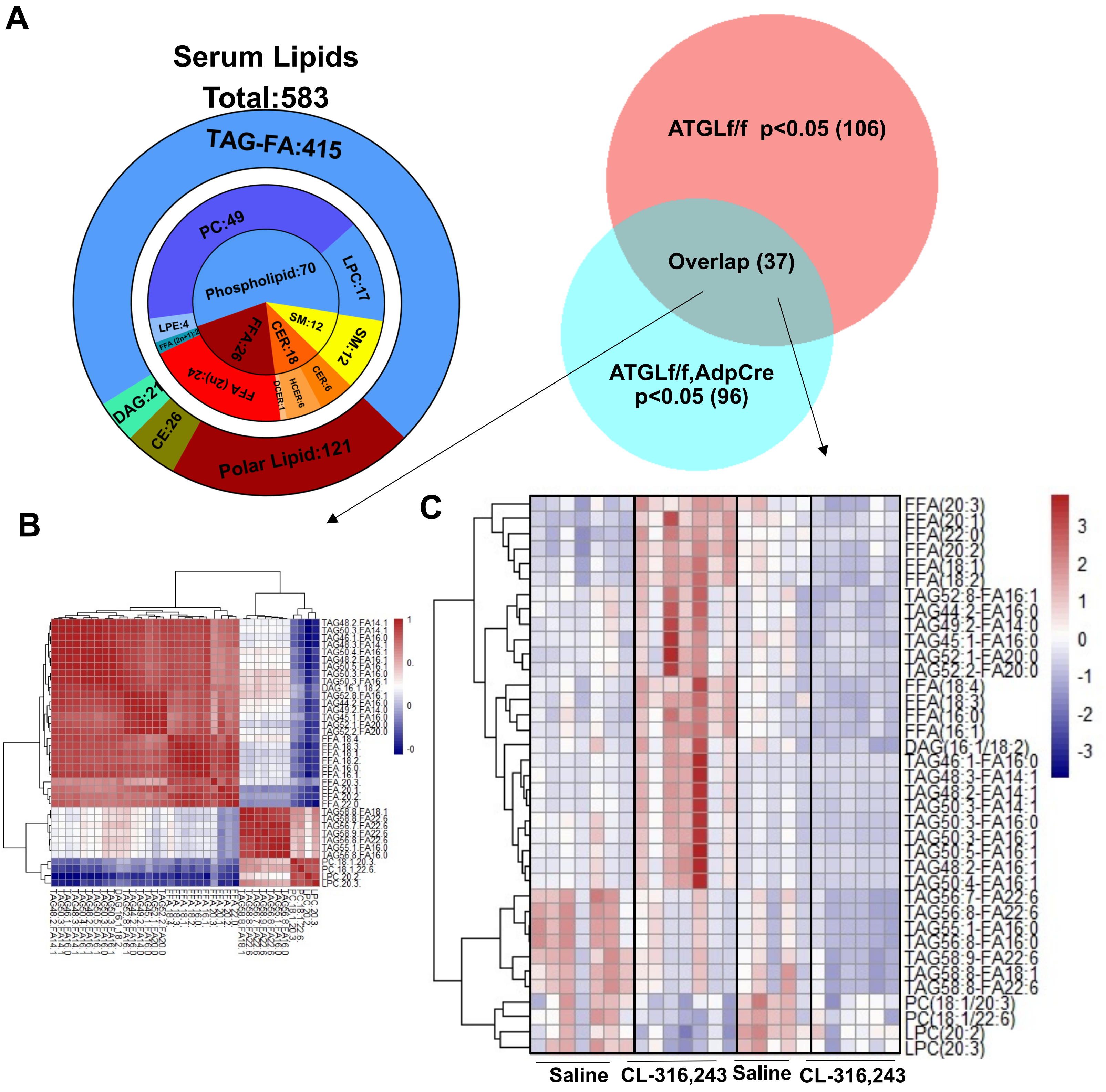

Supplement Figure 5. Significantly changed lipids, Overlapped between *Pnpla2$^{F/F}$* mice and *Pnpla2$^{F/F}$::Adipoq^{CRE}* mice.

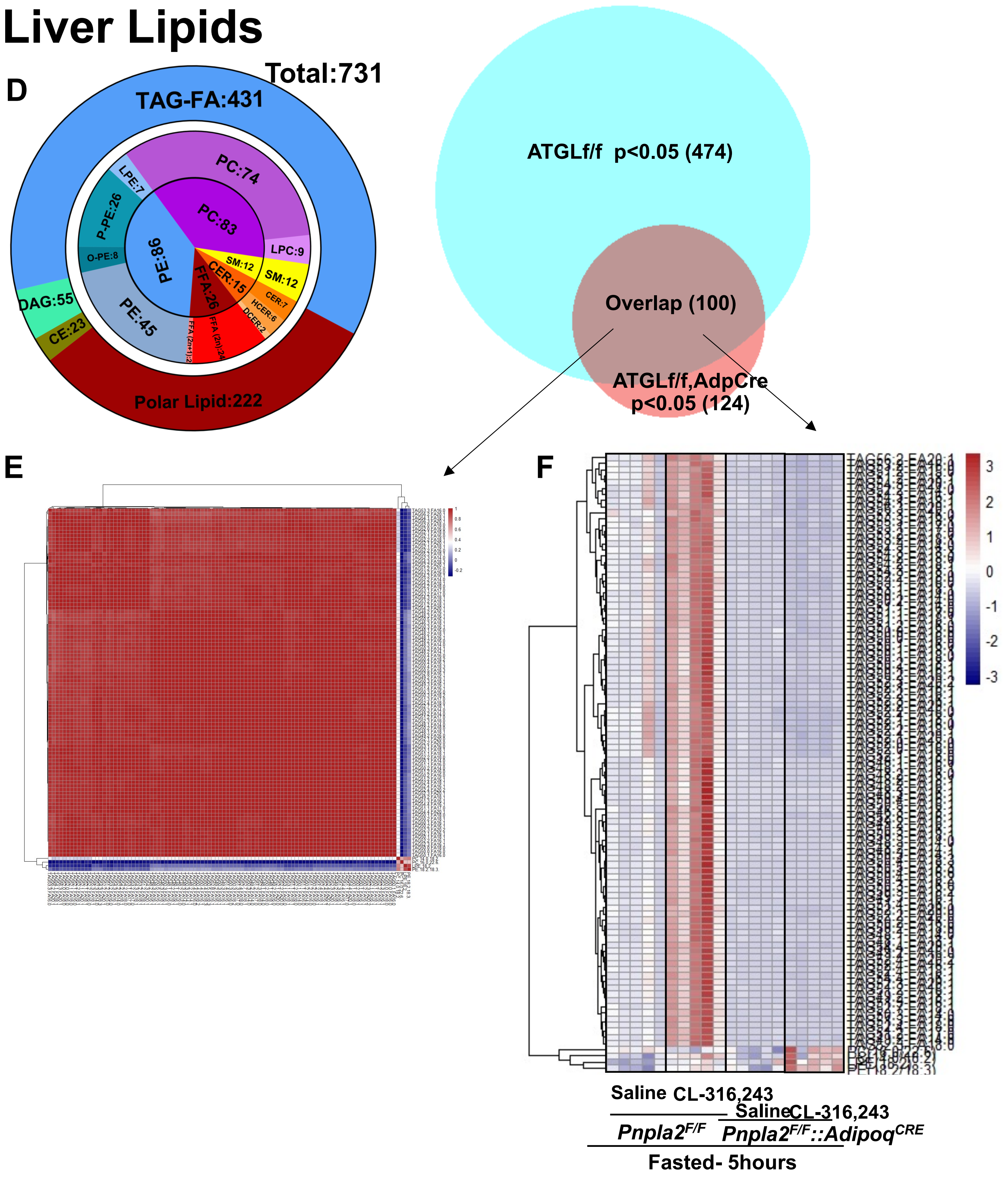

Supplement: Supplemental figure 5 — Comparative analysis of serum lipids from Pnpla2F/F mice and Pnpla2F/F::AdipoqCRE mice. A. Pie graph showing serum lipids grouped by the corresponding lipid classes. 583 serum lipids were identified. B. Overlapped Significant CL-induced and CL-reduced serum lipids between between Pnpla2F/F mice and Pnpla2F/F::AdipoqCRE mice. Correlation cluster of 37 overlapped significant lipids. C. Heatmap for all four groups in overlapped serum lipids. D. Pie graphic represents mice liver lipid molecular species grouped by the corresponding lipid classes. Totally dentified 583 lipids in serum lipids. E. Overlapped Significant CL-induced and CL-reduced serum lipids between Pnpla2F/F mice and Pnpla2F/F::AdipoqCRE mice. F. Heatmap for all four groups in overlapped hepatic lipids. [file mmc5.pdf]
