# Supplementary material for: Acute activation of adipocyte lipolysis reveals dynamic lipid remodeling of the hepatic lipidome
Source: J Lipid Res. 2023 Aug 26;65(2):100434. doi: 10.1016/j.jlr.2023.100434 (PMC10839691; doi:10.1016/j.jlr.2023.100434)

Supplement Figure 7. The concentration of specific lipid species in the liver after 5hrs CL administration by LC-MS

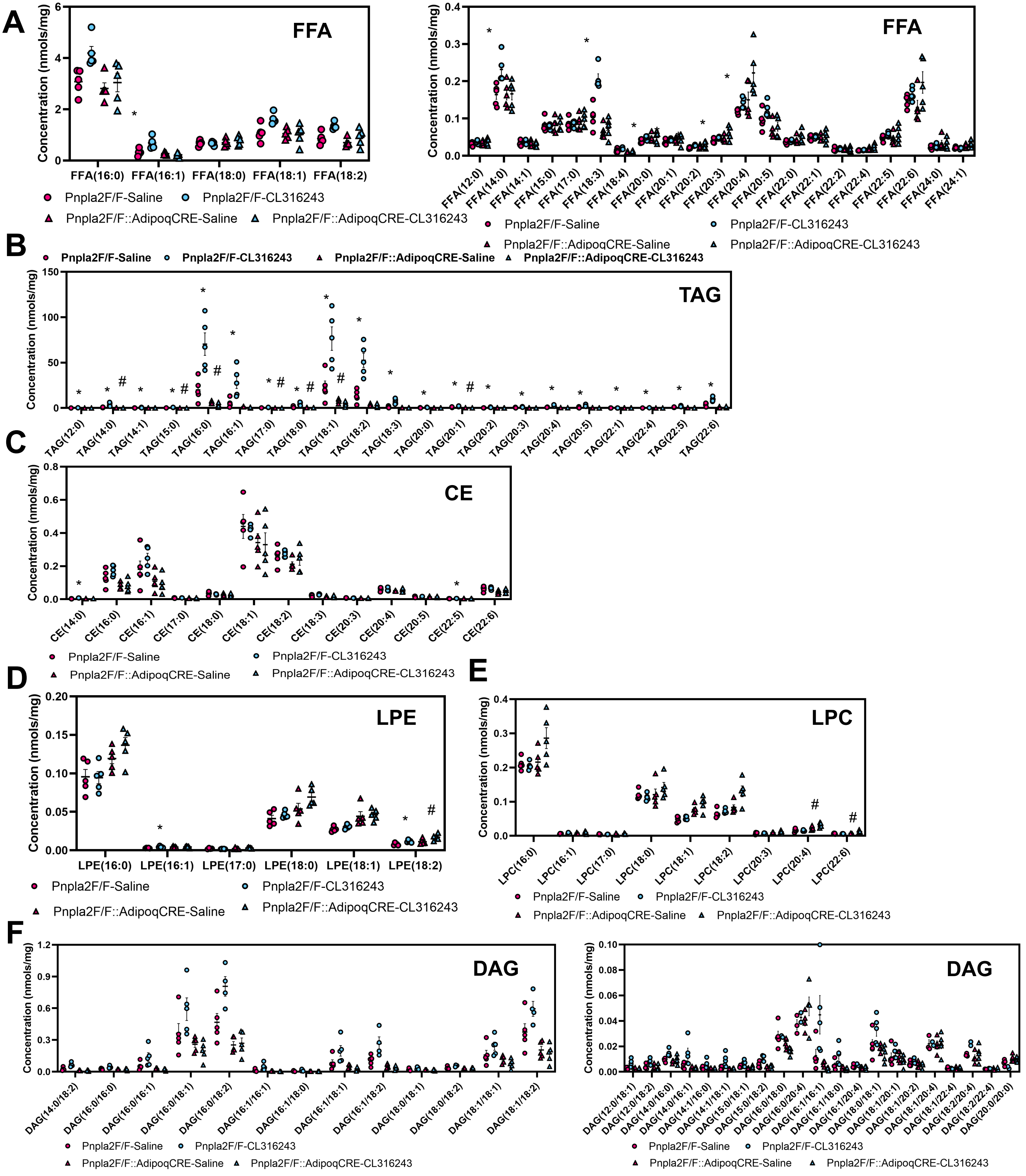

Supplement Figure 7. The concentration of specific lipid species in the liver after 5hrs CL administration by LC-MS

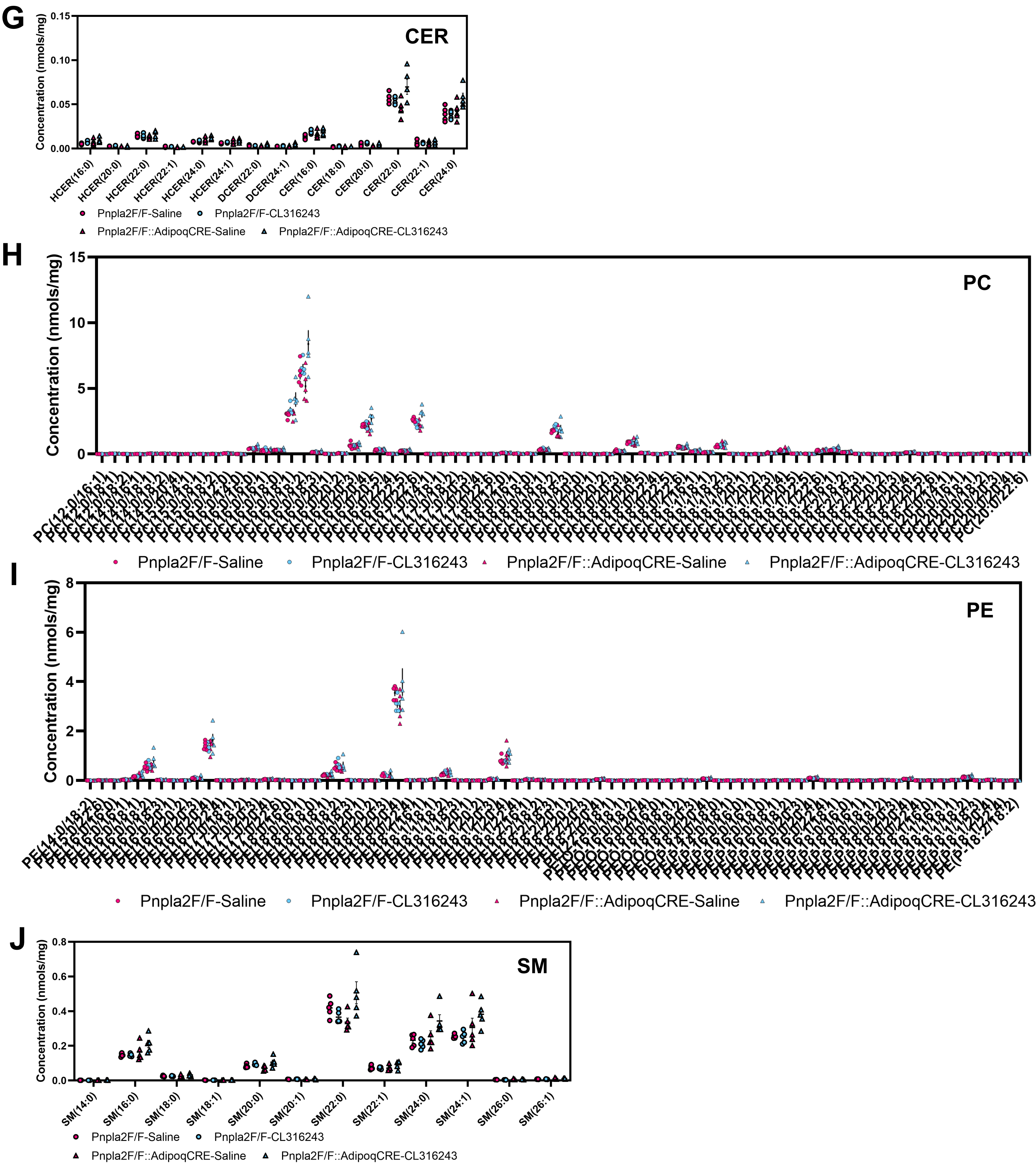

Supplement: Supplemental figure 7 — Quantitative analysis of liver lipids in Pnpla2F/F and Pnpla2F/F::AdipoqCRE mice after 5 h CL administration. Mice were administered a single dose of Saline or 1mg/kg CL-316,243 and sacrificed after 5 hours (n = 5–7). [file mmc7.pdf]
