# Supplementary material for: Acute activation of adipocyte lipolysis reveals dynamic lipid remodeling of the hepatic lipidome
Source: J Lipid Res. 2023 Aug 26;65(2):100434. doi: 10.1016/j.jlr.2023.100434 (PMC10839691; doi:10.1016/j.jlr.2023.100434)

Supplement Figure 9. The concentration of specific lipid species in the condition medium after vehicle or CL administration by LC-MS.

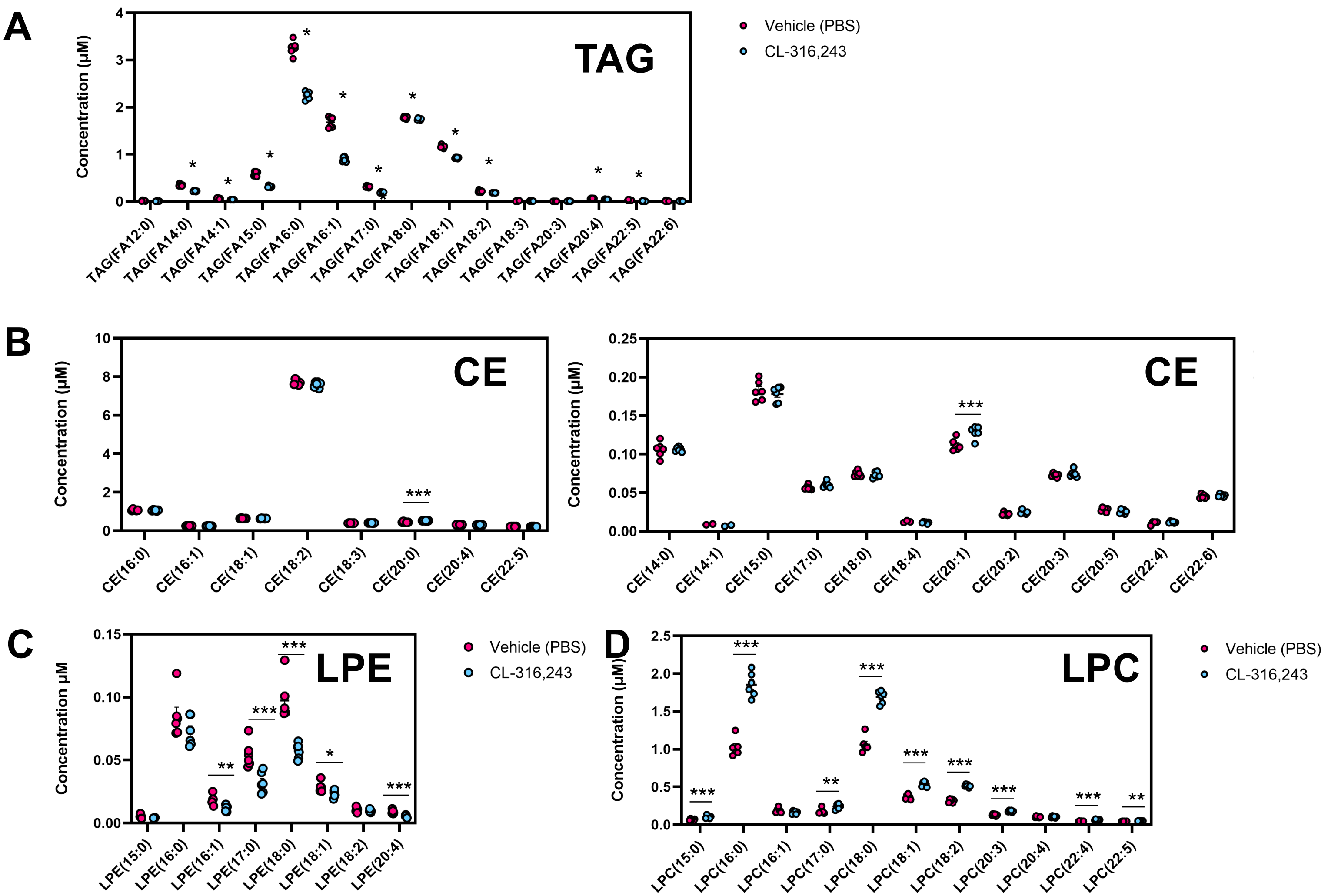

Supplement Figure 9. The concentration of specific lipid species in the condition medium after vehicle or CL administration by LC-MS.

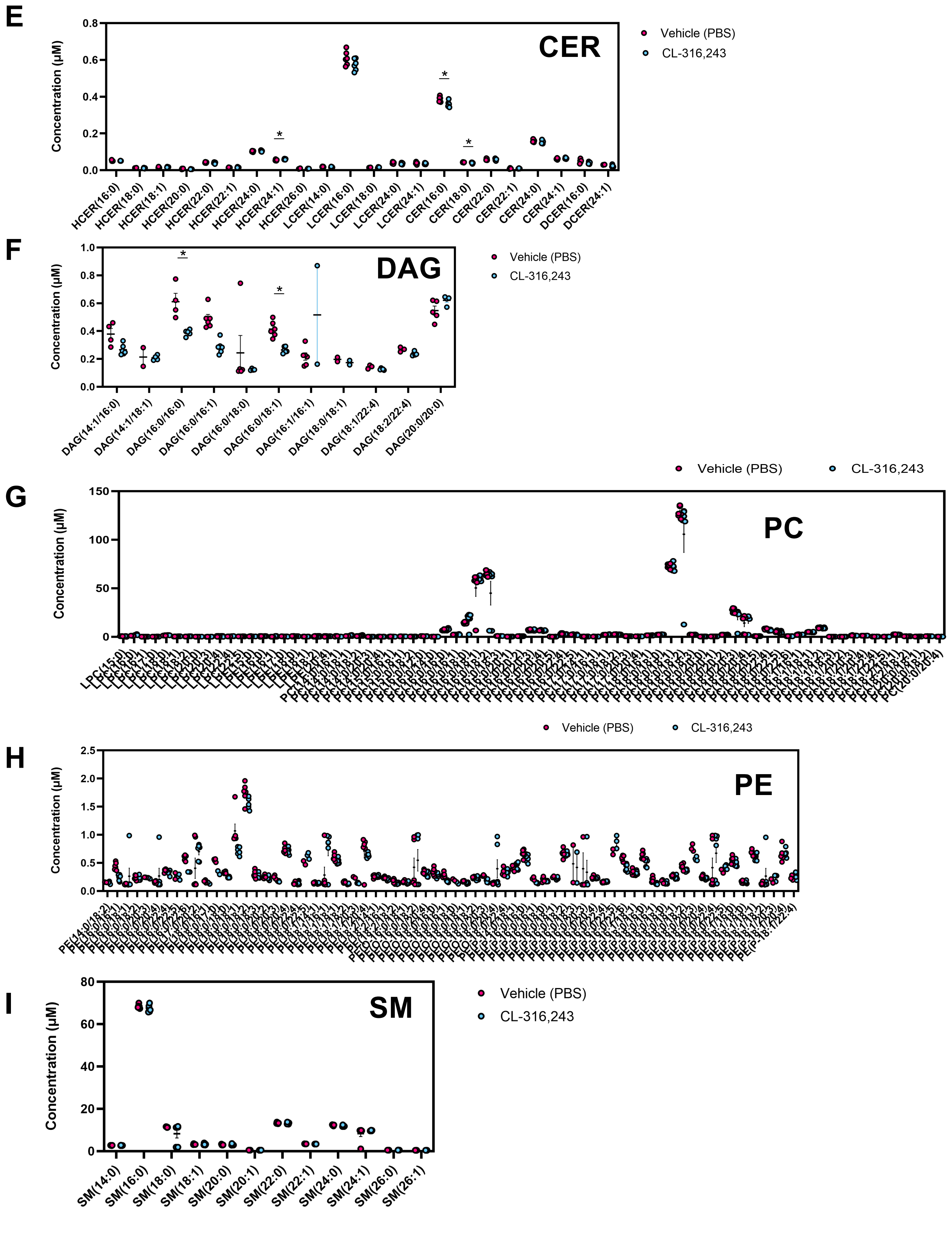

Supplement: Supplemental figure 9 — Quantitative lipid analysis of condition medium of differentiated adipocytes after vehicle or CL-316,243 administration. (n = 6, ∗: P < 0.05, ∗∗: P < 0.01, ∗∗∗: P < 0.005). [file mmc9.pdf]
