# Supplementary material for: Acute activation of adipocyte lipolysis reveals dynamic lipid remodeling of the hepatic lipidome
Source: J Lipid Res. 2023 Aug 26;65(2):100434. doi: 10.1016/j.jlr.2023.100434 (PMC10839691; doi:10.1016/j.jlr.2023.100434)
Supplement: Cover Art — Surface of the adipose tissue using light microscopy. The image of the adipose tissue reflects the mass spec analysis of products of adipose tissue lipolysis highlighted in Zhang et al. Image was captured by Julia Mack and tissue was processed by Mirian Krystel de Siqueira. [file mmc11.docx]

Surface of the adipose tissue using light microscopy. The image of the adipose tissue reflects the mass spec analysis of products of adipose tissue lipolysis highlighted in Zhang et al. Image was captured by Julia Mack and tissue was processed by Mirian Krystel de Siqueira.
